# InCl_3_-Catalyzed One-Pot Synthesis of Pyrrolo/Indolo- and Benzooxazepino-Fused Quinoxalines

**DOI:** 10.1021/acsomega.4c05239

**Published:** 2024-07-16

**Authors:** Nuray Esra Aksakal, Metin Zora

**Affiliations:** † Department of Chemistry, Faculty of Arts and Science, Middle East Technical University, 06800 Ankara, Turkey; ‡ Department of Nutrition and Dietetics, Faculty of Health Sciences, Halic University, 34060 Istanbul, Turkey

## Abstract

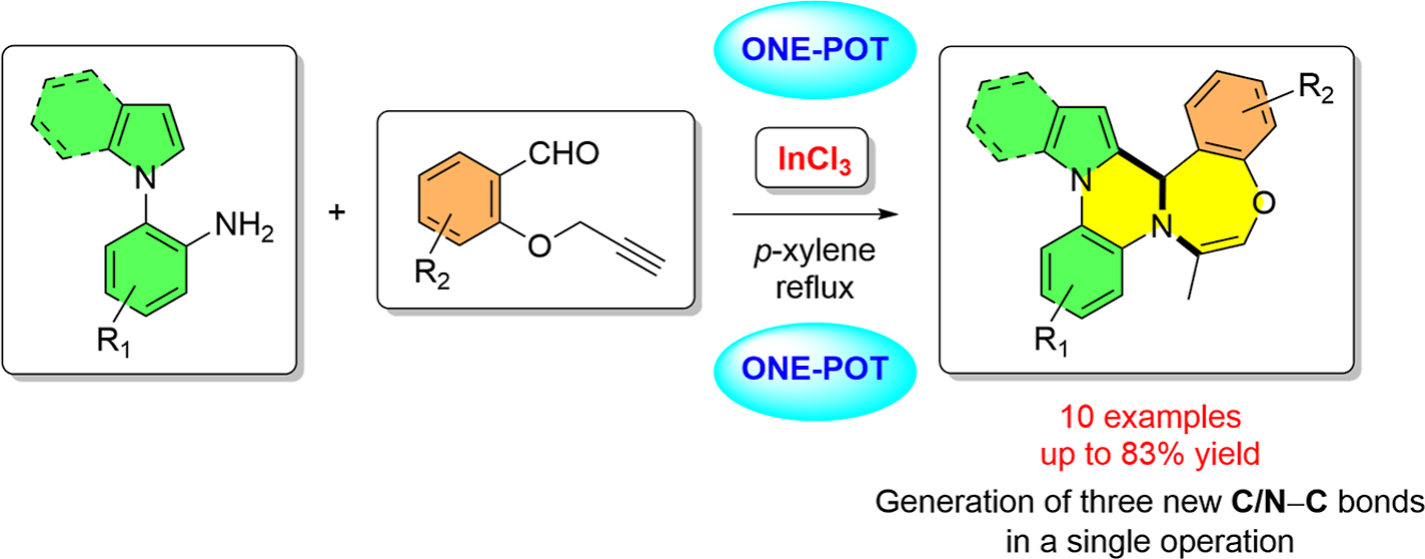

In this paper, we describe an efficient InCl_3_-catalyzed two-component reaction of 1-(2-aminophenyl)pyrroles/indoles and 2-propargyloxybenzaldehydes for the direct synthesis of 12b*H*-benzo[6,7]1,4-oxazepino[4,5-*a*]pyrrolo/indolo[2,1-*c*]quinoxalines. This high atom- and step-economical one-pot process generates three new C/N–C bonds in a single synthetic operation, resulting in the formation of new six- and seven-membered heterocyclic rings. The easy availability of the starting materials, the use of the relatively inexpensive indium catalyst, and the good substrate scope are the salient features of this strategy. The proposed mechanistic pathway involves imine formation, two consecutive cyclizations via electrophilic aromatic substitution and nucleophilic addition reactions, and the H shift step.

## Introduction

**Scheme 1:**
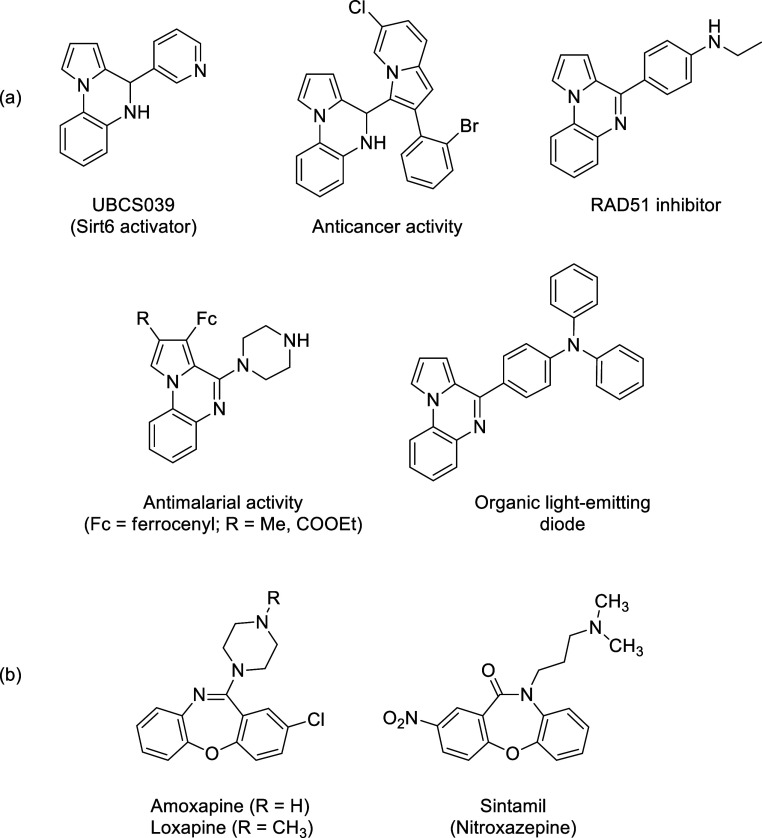
Representative Examples of Pyrroloquinoxalines (a) and Benzoxazepines (b)

Quinoxalines, as an important class of heterocyclic molecules, are attractive structural leads in medicinal chemistry due to their capacity to provide biological responses against various diseases.^1^ In fact, quinoxalines, called also benzopyrazines, have been the focus of a large number of investigations for years in the design and synthesis of novel biologically active agents that exhibit remarkable biological activities.^2^ Quinoxaline derivatives have been reported to possess a wide range of medicinal activities, including antibacterial, antidiabetic, anti-inflammatory, antimicrobial, antithrombotic, antitumor, and antiviral, and various enzyme inhibitory and receptor antagonist properties.^3^ Among quinoxaline derivatives, pyrrolo[1,2-*a*]quinoxalines and their partly hydrogenated derivatives, such as 4,5-dihydropyrrolo[1,2-*a*]quinoxalines, have received considerable attention since they are often found in the structures of many biologically active compounds and functional molecules.^4^ In recent years, pyrrolo[1,2-*a*]quinoxalines and derivatives have been extensively studied since they exhibit a plethora of biological activities, such as antifungal,^5^ antileishmanial,^6^ antiparasitic and antimalarial,^7^ antimycobacterial,^8^ anti-HCV and anti-HIV,^9^ antituberculosis,^10^ antiulcer,^11^ and antiproliferative and anticancer properties.^12^ These compounds have also been reported as inhibitors of enzymes FAAH and MAGL^13^ and human protein kinases CK2 and RAD51.^12^ In addition, they act as 5-HT_3_ receptor agonists^14^ and central dopamine,^15^ cannabinoid type 1 ,^16^ and glucagon receptor antagonists.^17^ Moreover, they show fluorescent and nonlinear optical properties, which lead to their applications in fluorescent probes and optical devices.^18^ Some examples of the important pyrrolo[1,2-*a*]quinoxalines are given in Scheme 1a.

Over the years, numerous methods have been developed for the synthesis of pyrrolo[1,2-*a*]quinoxalines, and new variants continue to appear. Pyrrolo[1,2-*a*]quinoxalines are generally synthesized from pyrroles or quinoxalines or from the compounds that are not initially derivatives of pyrroles or quinoxalines.^4^ In this regard, 1-(2-aminophenyl)pyrrole (2-(1*H*-pyrrol-1-yl)aniline) (**1**) and derivatives have emerged as valuable substrates since their cyclocondensation with carbonyl compounds such as aldehydes and ketones provided a diverse array of pyrrolo[1,2-*a*]quinoxalines and/or their dihydro derivatives.^19^ Notably, the use of functionally substituted aldehydes and ketones in these reactions may lead to the formation of new heterocyclic systems.

1,4-Oxazepines represent a privileged class of seven-membered nitrogen- and oxygen-containing heterocyclic compounds^20,21^ as they appear in the structures of many bioactive molecules and pharmaceutical compounds.^22^ In fact, 1,4-oxazepines are frequently utilized to cure various diseases such as allergic bronchitis and related asthma,^23^ epilepsy and trigeminal neuralgia,^24^ breast cancer,^25^ and psychotic disorders.^26^ Among 1,4-oxazepine derivatives, benzo-fused derivatives, commonly known as benzoxazepines, have gained more importance in the design and synthesis of novel bioactive heterocyclic molecules that display remarkable pharmacological and biological properties.^27^ The major medicinal properties of benzoxazepines include analgesic,^28^ antiallergic,^29^ antibacterial,^30^ anticonvulsant,^31^ antidepressant,^32^ antihistaminic,^33^ antiinflammatory,^34^ antipsychotic,^35^ anxiolytic,^36^ antiulcer,^37^ and antitumor^38^ activities. Some examples of the benzoxazepine-containing drugs are given in Scheme 1b. Amoxapine^39^ and Sintamil (nitroxazepine)^40^ display antidepressant properties, while Loxapine^41^ shows antipsychotic and antischizophrenic activities.

Recently, hybrid molecules, in which two or more pharmacophores are combined together in one molecule, have garnered significant interest since they provide enhanced or unusual activities as compared to their individual counterparts.^42,43^ Notably, heterocyclic hybrid molecules have exhibited better specificity, patient compliance, and aptitude to overcome drug resistance and reduced side effects.^44^ An ever-continuing aspect of these studies is to find novel hybrid molecules that will provide a new mode of action for the treatment of a specific disease. In this regard, the combination of pyrrole/indole, quinoxaline, and 1,4-oxazepine units in one molecule may lead to the discovery of hybrid compounds with increased activity profile as compared to the parent compounds.

**Scheme 2:**
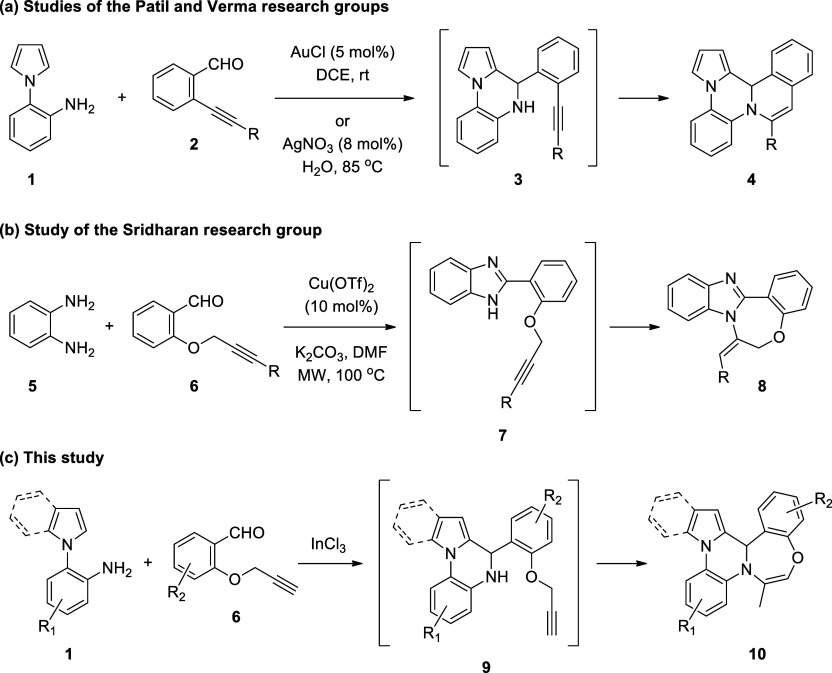
Strategies for the Synthesis of Fused Quinoxalines and 1,4-Oxazepines

The Patil and Verma research groups have recently reported that the reaction of 1-(2-aminophenyl)pyrrole (2-(1*H*-pyrrol-1-yl)aniline) (**1**) with 2-alkynylbenzaldehydes **2** under gold or silver catalysis has yielded isoquinolino-fused pyrrolo[2,1-*c*]quinoxalines **4** via intermediacy of 4-(2-ethynylphenyl)-4,5-dihydro-pyrrolo[1,2-*a*]quinoxaline **3**, which has undergone nucleophilic cyclization to furnish the final product (Scheme 2a).^45^ In recent times, 2-propargyloxybenzaldehydes, such as **6**, have emerged as valuable synthons in organic synthesis since, when reacted with proper compounds, they can give rise to the formation of 1,4-oxazepine derivatives. Sridharan and co-workers have demonstrated that Cu-catalyzed reaction between *o*-phenylenediamine (**5**) and 2-propargyloxybenzaldehyde **6** under microwave irradiation has led to in situ formation of 2-(2-propargyloxyphenyl)-1*H*-benzoimidazole **7**, which has immediately experienced an intramolecular cyclization to deliver benzoimidazo-fused 1,4-oxazepines **8** (Scheme 2b).^46^ Our continued interest in the synthesis of new heterocyclic frameworks as potential pharmaceuticals and scaffolds has prompted us to investigate the InCl_3_-catalyzed reaction of 1-(2-aminophenyl)pyrroles/indoles **1** with functionally substituted benzaldehydes, such as **6**. We have found that upon treatment with 2-propargyloxybenzaldehydes **6** in refluxing *p*-xylene under InCl_3_ catalysis, 1-(2-aminophenyl)pyrroles/indoles **1** have afforded 12b*H*-benzo[6,7]1,4-oxazepino[4,5-*a*]pyrrolo/indolo[2,1-*c*]quinoxalines **10** in a one-pot reaction, presumably via the intermediacy of 4-(2-(prop-2-yn-1-yloxy)phenyl)-4,5-dihydropyrrolo[1,2-*a*]quinoxaline **9** (Scheme 2c).^47^ To the best of our knowledge, the formation of such heterocyclic molecules from these reactions is without a precedent. In this paper, we report the preliminary results of this study.

## Results and Discussion

**Scheme 3:**
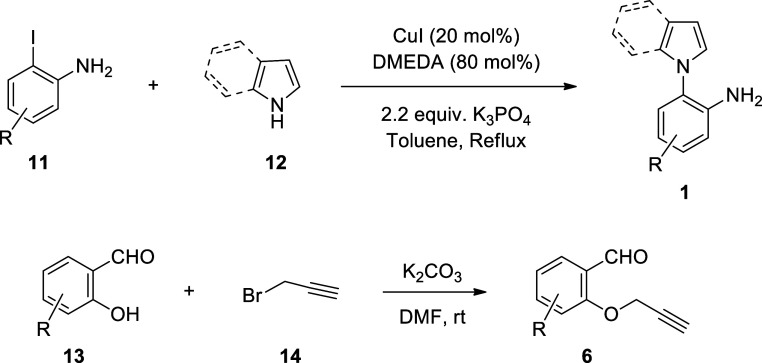
Synthesis of 1-(2-Aminophenyl)pyrroles/indoles and 2-Propargyloxybenzaldehydes

Initially, we synthesized the starting materials according to known literature procedures (Scheme 3). CuI-catalyzed coupling of iodoaniline derivatives **11** with pyrrole/indole (**12**) in the presence of DMEDA afforded the corresponding 1-(2-aminophenyl)pyrroles/indoles **1**.^48^ On the other hand, the S_N_2 reaction of salicylaldehyde (2-hydroxybenzaldehyde) derivatives **13** with propargyl bromide (**14**) in the presence of K_2_CO_3_ yielded 2-propargyloxybenzaldehydes **6**.^49^ For the identity of R groups and the yields of products **1** and **6**, see Supporting Information.

Subsequently, we made a short optimization study on the basis of our previous and ongoing studies (Table 1).^47,50^ We performed the reactions in the presence of a 2 mol % InCl_3_ catalyst since InCl_3_ is quite effective as a π-Lewis acid for the activation of alkyne moieties toward nucleophilic addition, which enables cyclization via carbon/nitrogen–carbon bond formation.^51^ In addition, it is moisture compatible and plays an important role in organic synthesis,^52^ especially in the synthesis of heterocycles.^53^ Briefly, they may present exciting opportunities for the development of new approaches and strategies. Initially, we carried out the reaction of 1-(2-aminophenyl)pyrrole (**1a**) with 2-propargyloxybenzaldehyde (**6a**) in toluene by heating to reflux for 4 h, which yielded a new heterocyclic compound, namely, 12b*H*-benzo[6,7]1,4-oxazepino[4,5-*a*]pyrrolo[2,1-*c*]quinoxaline (**10a**) but in a low yield (26%) (Table 1, entry 1). However, the same reaction in *p*-xylene formed product **10a** in a higher yield (68%) even in a shorter time (2 h) (Table 1, entry 2). Obviously, the higher reaction temperature resulted in a higher yield of product **10a**. When the reaction was carried out for 8 and 12 h, the yield of the product increased slightly (70%) (Table 1, entries 3 and 4). On the other hand, the longer reaction time such as 24 h reduced the yield of the product somewhat (68%) (Table 1, entry 5). So, the generality of the reaction was demonstrated by refluxing *p*-xylene using 2 mol % InCl_3_ as the catalyst, which was monitored by routine TLC analysis.

**Table 1:**
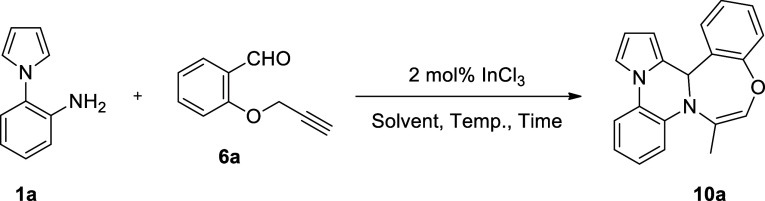
Optimization Studies for the Synthesis of 12b*H*-Benzo[6,7]1,4-oxazepino[4,5-*a*]pyrrolo/indolo[2,1-*c*]quinoxalines **10**^a^

entry	solvent	temp. (°C)	time (h)	yield (%)^b^
1	toluene	110	4	26
2	*p*-xylene	140	2	68
3	*p*-xylene	140	8	70
4	*p*-xylene	140	12	70
5	*p*-xylene	140	24	68

a Reactions were performed on a scale of 0.60 mmol of 1-(2-aminophenyl)pyrrole/indole **1**, 0.50 mmol of 2-propargyloxybenzaldehyde **6**, and 0.01 mmol of InCl_3_ in 10 mL of solvent under indicated conditions. For workup and purification, see Experimental Section.

b Isolated yield.

**Scheme 4:**
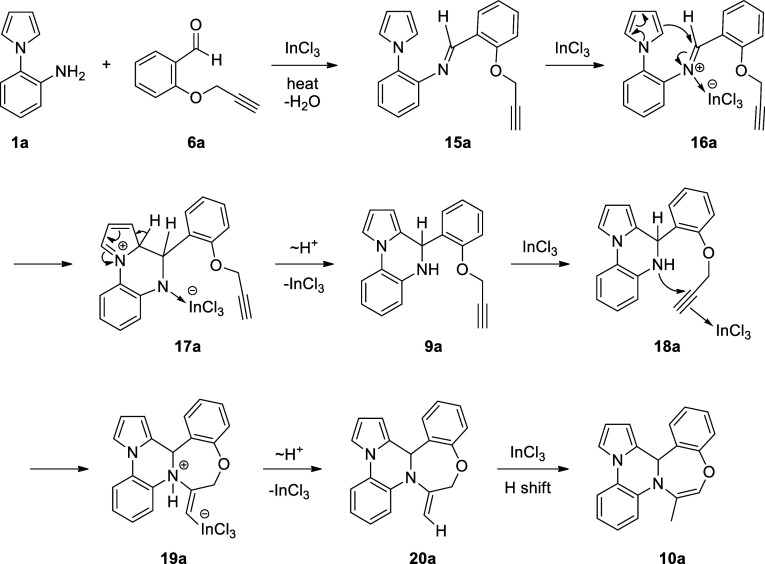
Mechanism Proposed for the Formation of 12b*H*-Benzo[6,7]1,4-oxazepino[4,5-*a*]pyrrolo/indolo[2,1-*c*]quinoxalines **10**

Having established the optimal reaction conditions, we turned our focus to investigate the scope and limitations of the methodology by employing a variety of 1-(2-aminophenyl)pyrroles/indoles **1** and 2-propargyloxybenzaldehydes **6** to access pyrrolo/indolo- and benzooxazepino-fused quinoxalines **10** (Table 2). All reactions proceeded smoothly and provided the expected products. Importantly, during the course of the reaction, three new C/N–C bonds were formed, which enabled the formation of new six- and seven-membered heterocyclic rings. Moreover, during the reaction, as it will be depicted in the mechanism of the reaction (Scheme 4), terminal alkyne carbon atom of **6** converted into a methyl carbon in the final product **10**. Therefore, the presence of the methyl peaks at 1.82–2.01 ppm in ^1^H NMR spectra and 16.7–17.4 ppm in ^13^C NMR spectra is clearly indicative of the formation of 12b*H*-benzo[6,7]1,4-oxazepino[4,5-*a*]pyrrolo/indolo[2,1-*c*]quinoxaline derivatives **10**. Overall, we synthesized 10 derivatives of quinoxalines **10**, the yields of which changed from 25 to 83%. Notably, the yields (47–83%) of pyrrolo-fused quinoxalines **10a–f** were mostly higher than those (25–50%) of indolo-fused quinoxalines **10g–j**. This might be the result of that in electrophilic aromatic substitution reactions; the 2-positions of pyrroles are clearly more reactive than the 2-positions of indoles owing to aromaticity concerns and resonance interactions.

**Table 2:**
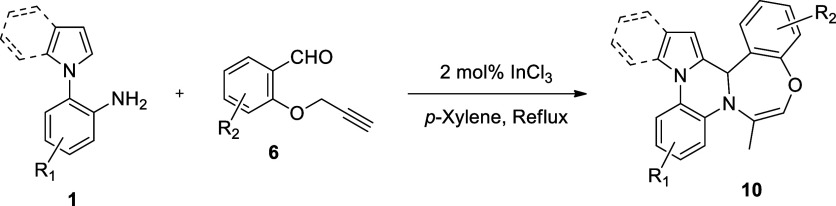

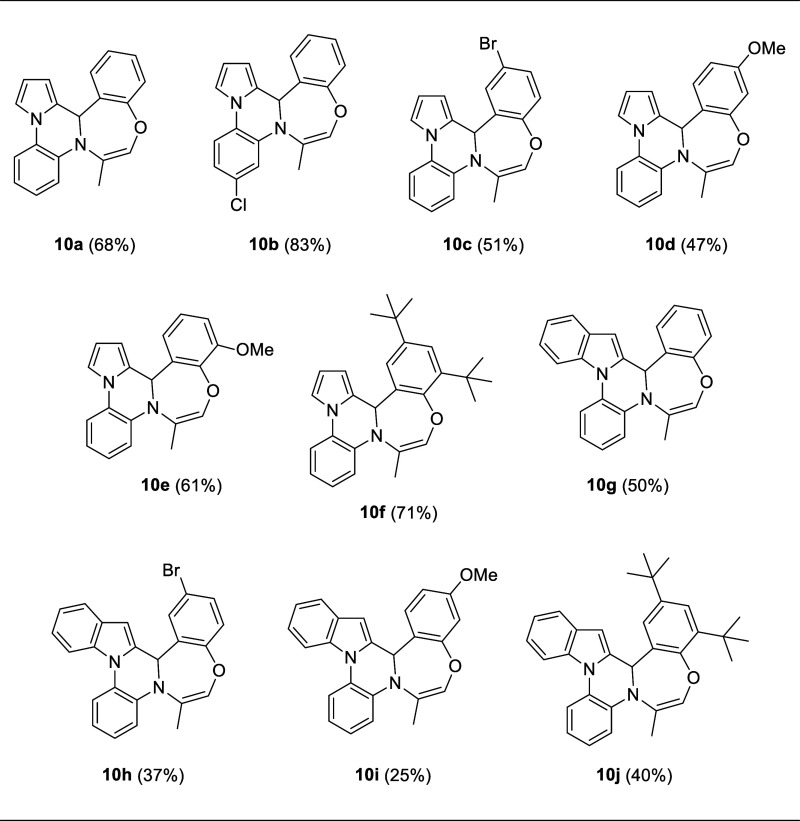
Synthesis of 12b*H*-Benzo[6,7]1,4-oxazepino[4,5-*a*]pyrrolo/indolo[2,1-*c*]quinoxalines **10**^a^^,^^b^

a Reactions were performed on a scale of 0.60 mmol of 1-(2-aminophenyl)pyrrole/indole **1**, 0.50 mmol of 2-propargyloxybenzaldehyde **6**, and 0.01 mmol of InCl_3_ in 10 mL of *p*-xylene at reflux. For workup and purification, see Experimental Section.

b Isolated yields.

A possible mechanism for the synthesis of pyrrolo/indolo- and benzooxazepino-fused quinoxalines **10** is shown for the reaction between 1-(2-aminophenyl)pyrrole **1a** and 2-propargyloxybenzaldehyde **6a** in Scheme 4. After the formation of imine **15a**, InCl_3_ activates the imine functionality through intermediate **16a**. This triggers the intramolecular cyclization through the electrophilic aromatic substitution reaction of the pyrrole moiety to afford intermediate **17a**, which upon aromatization yields pyrroloquinoxaline **9a**. Then, the coordination of the indium (III) to alkyne group generates in situ intermediate **18a**, in which the unsaturated alkyne moiety is activated toward nucleophilic addition. Afterward, nucleophilic addition of the amine group to the alkyne moiety in 7-*exo*-*dig* manner constructs the seven-membered ring (i.e., 1,4-oxazepine ring) of intermediate **19a**, which after subsequent deprotonation delivers heterocycle **20a**. Finally, the InCl_3_-catalyzed 1,3-H shift produces pyrrolo- and benzooxazepino-fused quinoxaline **10a**.

## Conclusions

In summary, we have established an InCl_3_-catalyzed two-component reaction between 1-(2-aminophenyl)pyrroles/indoles **1** and 2-propargyloxybenzaldehydes **6** for the synthesis of pyrrolo/indolo- and benzooxazepino-fused quinoxaline derivatives **10** in moderate to good yields (up to 83%). This operationally simple one-pot approach shows several advantages including high step- and atom-economy, generation of three new C/N–C bonds in a single synthetic process, good substrate scope, and the use of a relatively inexpensive indium catalyst. Mechanistically, the reaction proceeds via imine formation, successive intramolecular cyclizations through electrophilic aromatic substitution and nucleophilic addition reactions, and the H shift sequence. Importantly, the practical utility of the developed strategy and the skeletal diversity of the synthesized heterocyclic frame may provide many new nitrogen- and oxygen-based heterocyclic hybrid systems for drug discovery and development, which may modulate the activity of many targets that have been beyond the horizon of traditional compounds. Further investigation of the mechanism, scope, and limitations of this methodology is currently underway and will be reported in due course.

## Experimental Section

### General Information

^1^H and ^13^C NMR spectra were obtained at 400 and 100 MHz, respectively. Chemical shifts are given in parts per million (ppm) relative to CDCl_3_ (7.26 ppm in ^1^H NMR and 77.16 ppm in ^13^C NMR). Coupling constants (*J*) were given in hertz (Hz), and spin multiplicities were depicted by the following symbols: s (singlet), d (doublet), t (triplet), q (quartet), and m (multiplet). Infrared (IR) spectra were recorded by using attenuated total reflection. Peaks diagnostic for major functional groups were listed in reciprocal centimeters (cm^–1^). Mass spectra (MS) and high-resolution MS (HRMS) were obtained using electrospray ionization (ESI) with micro-Tof; *m*/*z* values are reported (for each measurement, the mass scale was recalibrated with sodium formate clusters, and samples were dissolved and measured in MeOH or CH_3_CN). Flash chromatography was performed with “flash-grade” silica gel (230–400 mesh) in thick-walled glass columns. TLC was realized by commercially available 0.25 mm silica gel plates, and visualization was achieved with a short-wavelength UV lamp (254 nm). The relative proportions of solvents used in chromatography indicate the volume/volume ratio. Unless otherwise stated, all commercially available reagents were used directly without purification. All solvents used in chromatography and reactions were distilled or dried properly for purity. The inert atmosphere was created using a slight positive pressure (ca. 0.1 psi) of argon. All glassware was dried in an oven prior to use.

1-(2-Aminophenyl)pyrroles/indoles (pyrrole/indole-substituted anilines) **1** and *o*-propargyloxybenzaldehydes **6** were synthesized according to literature studies (see Supporting Information).^48,49^

### General Procedure

#### Synthesis of Pyrrolo/Indolo- and Benzooxazepino-Fused Quinoxalines **10** (Table 2)

The corresponding *o*-propargyloxybenzaldehyde **6** (0.5 mmol) was dissolved in *p*-xylene (10 mL), and the proper pyrrole/indole-substituted aniline **1** (0.6 mmol) was added in one portion under argon. After the reaction mixture was stirred for half an hour, InCl_3_ (0.01 mmol) was added. The resulting reaction mixture was then refluxed at 140 °C for approximately 2 h. (The progress of the reaction was monitored by routine TLC analysis.) After the reaction was over, *p*-xylene was removed under reduced pressure. Purification of the obtained crude product by flash column chromatography on silica gel using 19:1 hexane/ethyl acetate as the eluent afforded the corresponding quinoxaline derivative **10**.

#### 6-Methyl-12b*H*-benzo[6,7][1,4]oxazepino[4,5-*a*]pyrrolo[2,1-*c*]quinoxaline (**10a**)

The general procedure was followed using 2-(prop-2-ynyloxy)benzaldehyde (**6a**) (0.1 g, 0.6 mmol), 2-(1*H*-pyrrol-1-yl)aniline (**1a**) (48) (120 mg, 0.8 mmol), and InCl_3_ (2.2 mg, 0.01 mmol). Purification of the crude product by flash column chromatography on silica gel afforded 128.5 mg (68% yield) of the indicated product. ^1^H NMR (400 MHz, CDCl_3_): δ 7.40 (dd, *J* = 8.0, 1.6 Hz, 1H), 7.33 (m, 1H), 7.08 (td, *J* = 7.8, 1.2 Hz, 1H), 6.92 (m, 3H), 6.70 (t, *J* = 7.2 Hz, 1H), 6.66 (dd, *J* = 8.0, 1.2 Hz, 1H), 6.46 (t, *J* = 3.2 Hz, 1H), 6.43 (s, 1H), 6.30 (m, 2H), 6.19 (dd, *J* = 3.6, 1.6 Hz, 1H), 1.84 (d, *J* = 1.2 Hz, 3H); ^13^C NMR (100 MHz, CDCl_3_): δ 155.6, 134.7, 133.1, 132.9, 129.2, 127.7, 127.0, 126.0, 124.9, 122.7, 121.0, 120.2, 119.8, 117.7, 115.1, 114.7, 110.4, 105.9, 57.7, 17.0 (CH_3_); IR (neat): 3855, 3651, 2240, 2144, 2069, 2031, 1965, 1751, 1509, 1213, 1123, 761, 693, 562, 493, 459, 426, 406 cm^–1^; MS (ESI, *m*/*z*): 299.12 [M – H]^+^; HRMS (ESI): calcd for C_20_H_15_N_2_O, 299.1184 [M – H]^+^; found, 299.1162.

#### 3-Chloro-6-methyl-12b*H*-benzo[6,7][1,4]oxazepino[4,5-*a*]pyrrolo[2,1-*c*]quinoxaline (**10b**)

The general procedure was followed using 2-(prop-2-ynyloxy)benzaldehyde (**6a**) (0.1 g, 0.6 mmol), 5-chloro-2-(1*H*-pyrrol-1-yl)aniline (**1b**) (100.1 mg, 0.5 mmol), and InCl_3_ (2.2 mg, 0.01 mmol). Purification of the crude product by flash column chromatography on silica gel afforded 174.6 mg (83%) of the indicated product. ^1^H NMR (400 MHz, CDCl_3_): δ 7.44 (m, 2H), 7.28 (td, *J* = 7.6, 1.6 Hz, 1H), 7.11 (dd, *J* = 8.4, 1.2 Hz, 1H), 7.00 (dd, *J* = 8.8, 2.4 Hz, 1H), 6.91 (*J* = 7.6, 1.2 Hz, 1H), 6.80 (d, *J* = 2.0 Hz, 1H), 6.63 (t, *J* = 3.2 Hz, 1H), 6.57 (s, 1H), 6.49 (d, *J* = 1.2 Hz, 1H), 6.45 (dd, *J* = 7.6, 1.2 Hz, 1H), 6.36 (dd, *J* = 3.6, 1.2 Hz, 1H), 2.01 (d, *J* = 1.2 Hz, 3H). ^13^C NMR (100 MHz, CDCl_3_): δ 155.3, 135.3, 134.1, 132.7, 130.1, 129.4, 126.9, 126.3, 125.6, 122.9, 120.3, 120.0, 119.9, 117.4, 116.0, 114.8, 110.8, 106.3, 57.9, 16.7 (CH_3_); IR (neat): 3855, 1664, 1604, 1509, 1480, 1449, 1386, 1341, 1267, 1212, 1116, 990, 874, 810, 794, 765, 723, 695, 632, 550, 453 cm^–1^; MS (ESI, *m*/*z*): 333.08 [M – H]^+^; HRMS (ESI): calcd for C_20_H_14_ClN_2_O, 333.0795 [M – H]^+^; found, 333.0781.

#### 11-Bromo-6-methyl-12b*H*-benzo[6,7][1,4]oxazepino[4,5-*a*]pyrrolo[2,1-*c*]quinoxaline (**10c**)

The general procedure was followed using 5-bromo-2-(prop-2-ynyloxy)benzaldehyde (**6b**) (0.1 g, 0.4 mmol), 2-(1*H*-pyrrol-1-yl)aniline (**1a**) (79.7 mg, 0.5 mmol), and InCl_3_ (2.2 mg, 0.01 mmol). Purification of the crude product by flash column chromatography on silica gel afforded 81.2 mg (51% yield) of the indicated product. ^1^H NMR (400 MHz, CDCl_3_): δ 7.41 (dd, *J* = 8.0, 1.2 Hz, 1H), 7.33 (m, 1H), 7.18 (dd, *J* = 8.4, 2.4 Hz, 1H), 6.99 (td, *J* = 7.6, 1.2 Hz, 1H), 6.91 (td, *J* = 8.0, 1.2 Hz, 1H), 6.79 (d, *J* = 8.4 Hz, 1H), 6.67 (dd, *J* = 8.0, 1.2 Hz, 1H), 6.45 (s, 1H), 6.39 (m, 2H), 6.28 (d, *J* = 1.2 Hz, 1H), 6.19 (dd, *J* = 7.6, 2.8 Hz, 1H), 1.83 (d, *J* = 1.2 Hz, 3H). ^13^C NMR (100 MHz, CDCl_3_): δ 154.7, 135.3, 134.5, 132.5, 132.1, 129.7, 127.5, 125.0, 124.8, 121.7, 121.4, 120.6, 117.6, 115.4, 115.3, 115.1, 110.6, 106.3, 57.5, 16.8 (CH_3_); IR (neat): 1668, 1608, 1509, 1471, 1399, 1336, 1292, 1258, 1216, 1189, 1163, 1124, 1095, 871, 831, 784, 770, 741, 700, 605, 543, 516, 496, 462 cm^–1^; MS (ESI, *m*/*z*): 377.03 [M – H]^+^; HRMS (ESI): calcd for C_20_H_15_BrN_2_O, 377.0290 [M – H]^+^; found, 377.0278.

#### 10-Methoxy-6-methyl-12b*H*-benzo[6,7][1,4]oxazepino[4,5-*a*]pyrrolo[2,1-*c*]quinoxaline (**10d**)

The general procedure was followed using 4-methoxy-2-(prop-2-ynyloxy)benzaldehyde (**6c**) (0.1 g, 0.5 mmol), 2-(1*H*-pyrrol-1-yl)aniline (**1a**) (99.8 mg, 0.6 mmol), and InCl_3_ (2.2 mg, 0.01 mmol). Purification of the crude product by flash column chromatography on silica gel afforded 82.2 mg (47%) of the indicated product. ^1^H NMR (400 MHz, CDCl_3_): δ 7.37 (dd, *J* = 8.0, 1.2 Hz, 1H), 7.29 (m, 1H), 6.97 (td, *J* = 7.6, 1.6 Hz, 1H), 6.86 (td, *J* = 7.6, 1.2 Hz, 1H), 6.64 (dd, *J* = 8.0, 1.2 Hz, 1H), 6.48 (d, *J* = 2.4 Hz, 1H), 6.43 (t, *J* = 3.2 Hz, 1H), 6.33 (s, 1H), 6.28 (d, *J* = 1.2 Hz, 1H), 6.21 (m, 2H), 6.15 (dd, *J* = 7.6, 1.6 Hz, 1H), 3.67 (s, 3H), 1.83 (d, *J* = 0.8 Hz, 3H). ^13^C NMR (100 MHz, CDCl_3_): δ 160.1, 156.1, 134.4, 132.9, 127.48, 127.46, 126.1, 125.3, 124.7, 120.9, 120.0, 117.4, 114.9, 114.4, 110.2, 107.7, 105.7, 105.6, 57.3, 55.3, 16.8 (CH_3_); IR (neat): 1669, 1611, 1503, 1423, 1377, 1338, 1282, 1241, 1187, 1164, 1131, 1085, 1036, 980, 852, 801, 771, 749, 708, 637, 567, 487, 546 cm^–1^; MS (ESI, *m*/*z*): 329.13 [M – H]^+^; HRMS (ESI): calcd for C_21_H_17_N_2_O_2_, 329.1290 [M – H]^+^; found, 329.1273.

#### 9-Methoxy-6-methyl-12b*H*-benzo[6,7][1,4]oxazepino[4,5-*a*]pyrrolo[2,1-*c*]quinoxaline (**10e**)

The general procedure was followed using 3-methoxy-2-(prop-2-ynyloxy)benzaldehyde (**6d**) (0.1 g, 0.5 mmol), 2-(1*H*-pyrrol-1-yl)aniline (**1a**) (99.8 mg, 0.6 mmol), and InCl_3_ (2.2 mg, 0.01 mmol). Purification of the crude product by flash column chromatography on silica gel afforded 106.7 mg (61%) of the indicated product. ^1^H NMR (400 MHz, CDCl_3_): δ 7.38 (dd, *J* = 7.6, 1.2 Hz, 1H), 7.31 (m, 1H), 6.96 (td, *J* = 7.6, 1.2 Hz, 1H), 6.88 (td, *J* = 7.6, 1.2 Hz, 1H), 6.72 (dd, *J* = 8.4, 1.6 Hz, 1H), 6.64 (m, 2H), 6.48 (s, 1H), 6.44 (t, *J* = 3.2 Hz, 1H), 6.43 (d, *J* = 1.2 Hz, 1H), 6.18 (dd, *J* = 7.6, 1.2 Hz, 1H), 5.89 (dd, *J* = 7.6, 1.6 Hz, 1H), 3.83 (s, 3H), 1.83 (d, *J* = 0.8 Hz, 3H). ^13^C NMR (100 MHz, CDCl_3_): δ 149.8, 144.2, 134.8, 134.5, 132.8, 127.8, 126.2, 124.8, 122.7, 121.4, 120.3, 118.7, 117.8, 115.0, 114.6, 112.2, 110.4, 105.9, 57.5, 56.3, 16.9 (CH_3_); IR (neat): 2836, 1671, 1583, 1507, 1474, 1381, 1337, 1255, 1205, 1179, 1123, 1070, 987, 928, 811, 777, 753, 670, 607 cm^–1^; MS (ESI, *m*/*z*): 329.13 [M – H]^+^; HRMS (ESI): calcd for C_21_H_17_N_2_O_2_, 329.1290 [M – H]^+^; found, 329.1267.

#### 9,11-Di-*tert*-butyl-6-methyl-12b*H*-benzo[6,7][1,4]oxazepino[4,5-*a*]pyrrolo[2,1-*c*]quinoxaline (**10f**)

The general procedure was followed using 3,5-di-*tert*-butyl-2-hydroxybenzaldehyde (**6e**) (0.1 g, 0.4 mmol), 2-(1*H*-pyrrol-1-yl)aniline (**1a**) (69.5 mg, 0.4 mmol), and InCl_3_ (2.2 mg, 0.01 mmol). Purification of the crude product by flash column chromatography on silica gel afforded 108.8 mg (71%) of the indicated product. ^1^H NMR (400 MHz, CDCl_3_): δ 7.37 (dd, *J* = 8.0, 1.6 Hz, 1H), 7.32 (dd, *J* = 2.8, 1.6 Hz, 1H), 7.10 (d, *J* = 2.4 Hz, 1H), 6.93 (td, *J* = 8.0, 1.6 Hz, 1H), 6.86 (td, *J* = 7.6, 1.2 Hz, 1H), 6.57 (dd, *J* = 8.0, 1.6 Hz, 1H), 6.47 (s, 1H), 6.44 (t, *J* = 3.2 Hz, 1H), 6.35 (d, *J* = 1.2 Hz, 1H), 6.18 (dd, *J* = 3.2, 1.2 Hz, 1H), 6.05 (d, *J* = 2.4 Hz, 1H), 1.84 (d, *J* = 0.8 Hz, 3H), 1.39 (s, 9H), 1.01 (s, 9H). ^13^C NMR (100 MHz, CDCl_3_): δ 152.0, 144.6, 139.8, 134.1, 133.9, 133.1, 128.1, 127.1, 124.7, 123.2, 122.0, 121.8, 120.3, 118.0, 114.9, 114.3, 110.5, 105.6, 57.4, 34.9, 34.4, 31.3, 30.4, 17.0 (CH_3_); IR (neat): 2960, 1508, 1475, 1334, 1253, 1215, 1138, 1088, 794, 773, 747, 697 cm^–1^; MS (ESI, *m*/*z*): 411.24 [M – H]^+^; HRMS (ESI): calcd for C_28_H_31_N_2_O, 411.2436 [M – H]^+^; found, 411.2427.

#### 7-Methyl-18b*H*-benzo[6,7][1,4]oxazepino[4,5-*a*]indolo[2,1-*c*]quinoxaline (**10g**)

The general procedure was followed using 2-(prop-2-ynyloxy)benzaldehyde (**6a**) (0.1 g, 0.6 mmol), 2-(1*H*-indol-1-yl)aniline (**1c**) (108.3 mg, 0.5 mmol), and InCl_3_ (2.2 mg, 0.01 mmol). Purification of the crude product by flash column chromatography on silica gel afforded 110.3 mg (50%) of the indicated product. ^1^H NMR (400 MHz, CDCl_3_): δ 8.14 (d, *J* = 8.0 Hz, 1H), 7.99 (m, 1H), 7.79 (d, *J* = 7.6 Hz, 1H), 7.41 (m, 1H), 7.33 (m, 1H), 7.12 (td, *J* = 8.0, 1.6 Hz, 1H), 7.05 (m, 2H), 7.00 (dd, *J* = 8.4, 1.2 Hz, 1H), 6.78 (m, 1H), 6.69 (td, *J* = 7.2, 1.2 Hz, 1H), 6.63 (s, 1H), 6.61 (s, 1H), 6.47 (dd, *J* = 7.6, 1.6 Hz, 1H), 6.41 (d, *J* = 1.2 Hz, 1H), 1.88 (d, *J* = 1.2 Hz, 3H). ^13^C NMR (100 MHz, CDCl_3_): δ 155.8, 134.7, 134.4, 134.3, 133.9, 131.9, 129.7, 129.3, 129.0, 127.1, 124.6, 122.8, 121.5, 121.4, 121.0, 120.9, 119.9, 118.2, 117.1, 112.0, 100.2, 58.4, 22.9, 17.3 (CH_3_); IR (neat): 2922, 1590, 1500, 1452, 1384, 1214, 1122, 744 cm^–1^; MS (ESI, *m*/*z*): 349.13 [M – H]^+^; HRMS (ESI): calcd for C_24_H_18_N_2_O, 349.1341 [M – H]^+^; found, 349.1329.

#### 2-Bromo-7-methyl-18b*H*-benzo[6,7][1,4]oxazepino[4,5-*a*]indolo[2,1-*c*]quinoxaline (**10h**)

The general procedure was followed using 5-bromo-2-(prop-2-ynyloxy)benzaldehyde (**6b**) (0.1 g, 0.4 mmol), 2-(1*H*-indol-1-yl)aniline (**1c**) (104.9 mg, 0.5 mmol), and InCl_3_ (2.2 mg, 0.01 mmol). Purification of the crude product by flash column chromatography on silica gel afforded 66.7 mg (37%) of the indicated product. ^1^H NMR (400 MHz, CDCl_3_): δ 8.10 (d, *J* = 8.0 Hz, 1H), 7.97 (m, 1H), 7.75 (d, *J* = 7.6 Hz, 1H), 7.39 (td, *J* = 7.0, 1.2 Hz, 1H), 7.29 (s, 1H), 7.18 (dd, *J* = 8.8, 2.4 Hz, 1H), 7.05 (m, 2H), 6.81 (d, *J* = 8.8 Hz, 1H), 6.75 (m, 1H), 6.57 (s, 1H), 6.52 (m, 2H), 6.33 (d, *J* = 1.2 Hz, 1H), 1.83 (d, *J* = 1.2 Hz, 3H). ^13^C NMR (100 MHz, CDCl_3_): δ 155.0, 134.5, 134.03, 134.0, 133.9, 133.3, 132.3, 129.7, 129.6, 128.8, 124.7, 123.0, 121.9, 121.6, 121.5, 121.44, 121.4, 118.2, 117.4, 115.5, 112.1, 100.7, 58.1, 17.1 (CH_3_); IR (neat): 1669, 1589, 1500, 1452, 1391, 1257, 1216, 1165, 1121, 1094, 825, 786, 743, 730, 665, 610, 521, 431 cm^–1^; MS (ESI, *m*/*z*): 427.04 [M – H]^+^; HRMS (ESI): calcd for C_24_H_17_BrN_2_O, 427.0446 [M – H]^+^; found, 427.0423.

#### 3-Methoxy-7-methyl-18b*H*-benzo[6,7][1,4]oxazepino[4,5-*a*]indolo[2,1-*c*]quinoxaline (**10i**)

The general procedure was followed using 4-methoxy-2-(prop-2-ynyloxy)benzaldehyde (**6c**) (0.1 g, 0.5 mmol), 2-(1*H*-indol-1-yl)aniline (**1c**) (132.3 mg, 0.6 mmol), and InCl_3_ (2.2 mg, 0.01 mmol). Purification of the crude product by flash column chromatography on silica gel afforded 50.4 mg (25%) of the indicated product. ^1^H NMR (400 MHz, CDCl_3_): δ 8.07 (d, *J* = 8.0 Hz, 1H), 7.92 (m, 1H), 7.71 (d, *J* = 8.0 Hz, 1H), 7.34 (m, 1H), 7.25 (m, 1H), 7.01 (m, 2H), 6.73 (m, 1H), 6.53 (s, 1H), 6.50 (d, *J* = 2.4 Hz, 1H), 6.47 (s, 1H), 6.33 (m, 1H), 6.29 (d, *J* = 8.8 Hz, 1H), 6.17 (dd, *J* = 8.4, 2.4 Hz, 1H), 3.66 (s, 3H), 1.83 (d, *J* = 1.2 Hz, 3H). ^13^C NMR (100 MHz, CDCl_3_): δ 156.6, 134.62, 134.6, 134.5, 133.9, 129.7, 127.8, 124.6, 124.2, 122.7, 121.5, 121.3, 121.2, 120.8, 118.2, 117.1, 112.0, 108.0, 105.9, 100.1, 58.1, 55.5, 17.4 (CH_3_); IR (neat): 3333, 2929, 1731, 1611, 1500, 1454, 1358, 1240, 1195, 1160, 1120, 1040, 734 cm^–1^; MS (ESI, *m*/*z*): 329.13 [M – H]^+^; HRMS (ESI): calcd for C_21_H_17_N_2_O_2_, 329.1290 [M – H]^+^; found, 329.1267.

#### 2,4-Di-*tert*-butyl-7-methyl-18b*H*-benzo[6,7][1,4]oxazepino[4,5-*a*]indolo[2,1-*c*]quinoxaline (**10j**)

The general procedure was followed using 3,5-di-*tert*-butyl-2-(prop-2-ynyloxy)benzaldehyde (**6e**) (0.1 g, 0.4 mmol), 2-(1*H*-indol-1-yl)aniline (**1c**) (91.8 mg, 0.4 mmol), and InCl_3_ (2.2 mg, 0.01 mmol). Purification of the crude product by flash column chromatography on silica gel afforded 68.4 mg (40%) of the indicated product. ^1^H NMR (400 MHz, CDCl_3_): δ 8.08 (d, *J* = 8.0 Hz, 1H), 7.91 (dd, *J* = 7.6, 2.0 Hz, 1H), 7.73 (d, *J* = 7.6 Hz, 1H), 7.35 (td, *J* = 8.4, 1.2 Hz, 1H), 7.27 (t, *J* = 6.0 Hz, 1H), 7.1 (d, *J* = 2.4 Hz, 1H), 6.98 (m, 2H), 6.61 (m, 2H), 6.57 (d, *J* = 2.4 Hz, 1H), 6.40 (d, *J* = 1.2 Hz, 1H), 6.20 (d, *J* = 2.4 Hz, 1H), 1.82 (d, *J* = 1.2 Hz, 3H), 1.4 (s, 9H), 0.94 (s, 9H); ^13^C NMR (100 MHz, CDCl_3_): δ 152.3, 145.0, 140.0, 132.9, 135.6, 134.6, 134.0, 130.0, 124.6, 123.2, 122.6, 122.4, 121.9, 121.6, 121.2, 121.0, 118.6, 117.0, 111.9, 100.1, 58.1, 34.9, 34.5, 31.3, 30.5, 17.3 (CH_3_); IR (neat): 2954, 1591, 1501, 1452, 1380, 1380, 1360, 1285, 1243, 1214, 1132, 881, 795, 772, 745, 726, 680, 435 cm^–1^; MS (ESI, *m*/*z*): 461.26 [M – H]^+^; HRMS (ESI): calcd for C_32_H_33_N_2_O, 461.2593 [M – H]^+^; found, 461.2569.

## Supporting Information Available

Experimental procedures, spectroscopic data, and/or copies of ^1^H and ^13^C NMR spectra for starting materials and products (PDF)

## Notes

This study has been partially abstracted from the *M. S. Thesis* of Nuray Esra Yazici (Aksakal) [Yazici, N. E., *M. S. Thesis*; Middle East Technical University (Ankara, Turkey), 2013].
