# Supplementary material for: InCl3-Catalyzed One-Pot Synthesis of Pyrrolo/Indolo- and Benzooxazepino-Fused Quinoxalines
Source: ACS Omega. 2024 Jul 16;9(30):33251–60. doi: 10.1021/acsomega.4c05239 (PMC11292660; doi:10.1021/acsomega.4c05239)
Supplement: Supplementary file 1 — ao4c05239_si_001.pdf [file ao4c05239_si_001.pdf]

$InCl_3$-Catalyzed One-Pot Synthesis of Pyrrolo/Indolo- and Benzooxazepino-Fused Quinoxalines

Nuray Esra Aksakal[a,b] and Metin Zora[a,*]

[a] Department of Chemistry, Faculty of Arts and Science, Middle East Technical University, 06800 Ankara, Turkey

[b] Department of Nutrition and Dietetics, Faculty of Health Sciences, Halic University, 34060 Istanbul, Turkey

Table of Contents

Experimental Section …………………………………………………………… S2

General information …………………………………………………………… S2

Table S1. Synthesis of pyrrole/indole-substituted anilines 1 ……………………… S3

General Procedure 1. Synthesis of pyrrole/indole-substituted anilines 1 …….. S3

Table S2. Synthesis of *o*-propargyloxybenzaldehydes 6 …………………………… S4

General Procedure 2. Synthesis of *o*-propargyloxybenzaldehydes 6 …………… S5

Copies of ^{1}H and ^{13}C NMR Spectra ………………………………………… S7-S24

References ……………………………………………………………………… S24

* Corresponding Author. E-mail address: zora@metu.edu.tr (M. Zora)

Experimental Section

General Information. ^1H and ^{13}C NMR spectra were recorded at 400 and 100 MHz, respectively. Chemical shifts were given in parts per million (ppm) relative to $CDCl_3$ (7.26 and 77.16 ppm in ^1H and ^{13}C NMR, respectively. Coupling constants (*J*) were given in hertz (Hz), and spin multiplicities were shown by the following symbols: s (singlet), d (doublet), t (triplet), q (quartet), and m (multiplet). Infrared (IR) spectra were obtained using attenuated total reflection (ATR). Band positions diagnostic for major functional groups were recorded in reciprocal centimeters (cm^{-1}). Mass spectra (MS) and high-resolution MS (HRMS) were obtained using electrospray ionization (ESI) with micro-Tof; *m/z* values are reported (for each measurement, the mass scale was recalibrated with sodium formate clusters, and samples were dissolved and measured in MeOH or CH_3CN). Flash chromatography was performed using thick-walled glass columns and "flash-grade" silica gel (230−400 mesh). TLC was accomplished by using commercially prepared 0.25 mm silica gel plates and visualization was effected with a short-wavelength UV lamp (254 nm). The relative proportions of solvents in chromatography solvent mixtures refer to the volume/volume ratio. All commercially available reagents were used directly without purification unless otherwise stated. All solvents used in reactions and chromatography were distilled and/or dried properly for purity. The inert atmosphere was created using slight positive pressure (ca. 0.1 psi) of argon. All glassware was dried in an oven prior to use.

1-(2-Aminophenyl)pyrroles/indoles (pyrrole/indole-substituted anilines) **1** and *o*-propargyloxybenzaldehydes **6** were synthesized according to literature studies (see below).[1-13]

Table S1. Synthesis of pyrrole/indole-substituted anilines **1**.

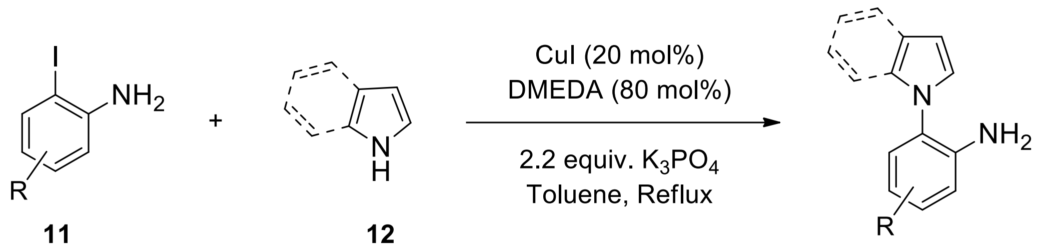

11 **12** **1**

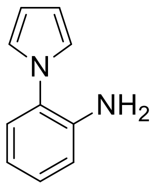

1a[a]

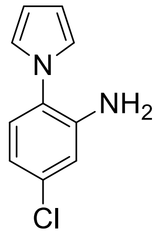

1b (55%)

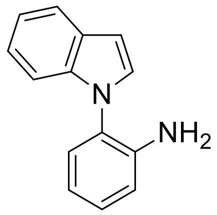

1c (80%)

[a] It is commercially available. So it was not synthesized.

General Procedure 1. Synthesis of pyrrole/indole-substituted anilines 1 (Table S1). The corresponding 2-iodoaniline **11** (0.5 mmol) was dissolved in toluene (15 ml). Then pyrrole/indole **12** (0.6 mmol) was added to the solution under argon. Next, *N*,*N*-dimethylethylenediamine (0.4 mmol), CuI (0.1 mmol) and K_3PO_4 (0.2 mol) were added to the flask, respectively. The resulting solution was refluxed at 110 °C for 24 h. After the reaction was over, solvent was evaporated under reduced pressure. Purification of the obtained crude product by flash chromatography on silica gel by using 19:1 hexane/ethyl acetate as the eluent afforded the corresponding pyrrole/indole-substituted aniline **1**.

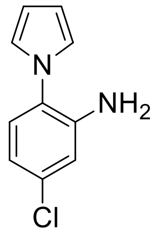

5-Chloro-2-(1*H*-pyrrol-1-yl)aniline (1b). General Procedure 1 was employed using 5-chloro-2-iodoaniline (**11b**) (100 mg, 0.4 mmol), pyrrole (**12a**) (31.8 mg, 0.5 mmol), *N*,*N*-dimethylethylenediamine (23.0 mg, 0.3 mmol), CuI (15.2 mg, 0.1 mmol) and K_3PO_4 (186.6 mg, 0.9 mmol). Purification of the crude product by flash column chromatography on silica gel afforded 42.2 mg (55%) of the indicated product. ^{1}H NMR (400 MHz, $CDCl_3$): δ 7.09 (d, *J* = 8.4 Hz, 1H), 6.83 (t, *J* = 2.4 Hz, 2H), 6.79 (m, 2H), 6.39 (t, *J* = 2.4 Hz, 2H), 3.79 (s, 2H); ^{13}C NMR (100 MHz, $CDCl_3$): δ 143.3,

134.1, 128.3, 126.1, 121.8, 118.3, 115.8, 109.9; IR (neat): 3312, 1618, 1507, 1321, 1243, 1069, 927, 854, 800, 738, 636, 537, 485, 456 cm^{-1}. The spectral data are in agreement with those reported previously for this compound.[1,2]

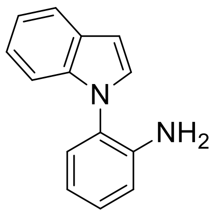

2-(1*H*-Indol-1-yl)aniline (1c). General Procedure 1 was employed using 2-iodoaniline (**11a**) (100 mg, 0.5 mmol), indole (**12b**) (64.1 mg, 0.6 mmol), *N*,*N*-dimethylethylenediamine (26.5 mg, 0.4 mmol), CuI (16.0 mg, 0.1 mmol) and K_3PO_4 (214.5 mg, 1.0 mmol). Purification of the crude product by flash column chromatography on silica gel afforded 76.5 mg (80%) of the indicated product. ^{1}H NMR (400 MHz, $CDCl_3$): δ 7.85 (m, 1H), 7.31 (m, 6H), 6.94 (td, J = 7.6, 1.2 Hz, 1H), 6.84 (d, J = 1.2 Hz, 1H), 6.82 (d, J = 3.2 Hz, 1H), 3.56 (s, 2H); ^{13}C NMR (100 MHz, $CDCl_3$): δ 143.2, 136.4, 129.2, 128.7, 128.63, 123.61, 124.8, 122.3, 121.0, 120.3, 118.5, 116.3, 110.8, 103.2; IR (neat): 3367, 1617, 1500, 1452, 1329, 1305, 1228, 1135, 1009, 954, 739, 494, 425 cm^{-1}. The spectral data are in agreement with those reported previously for this compound.[2-4]

Table S2. Synthesis of *o*-propargyloxybenzaldehydes **6**.

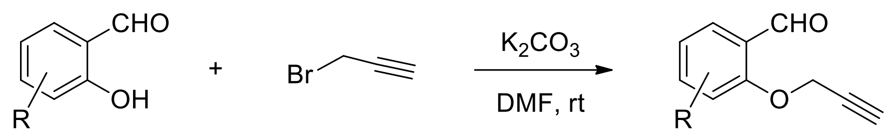

13 **14** **6**

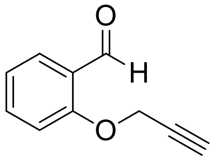

6a (85%)

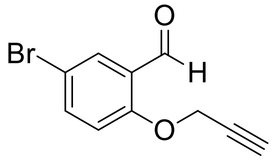

6b (85%)

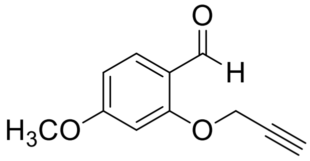

6c (80%)

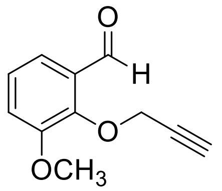

6d (81%)

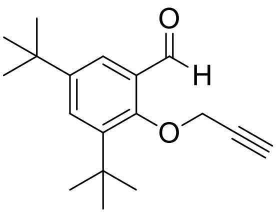

6e (83%)

General Procedure 2. Synthesis of *o*-propargyloxybenzaldehydes 6 (Table S2). The corresponding 2-hydroxybenzaldehyde **13** (0.8 mmol) was dissolved in approximately 20 ml of dry DMF and K_2CO_3 (113.1 mg, 0.8 mmol) was added to the solution under argon. Following the addition of base, propargyl bromide (**14**) (87.7 µl, 1.0 mmol) was added in one portion. Reaction was stirred at room temperature overnight. Then the content of reaction mixture was poured into a separatory funnel. Layers were separated and organic phase is washed with water (20 ml). Aqueous phase was further extracted with dichloromethane (3 x 20 ml). The collected organic layers were dried over $MgSO_4$ and evaporated on a rotary evaporator. Purification of the obtained crude product by flash chromatography on silica gel by using 19:1 hexane/ethyl acetate as the eluent afforded the corresponding o-propargyloxybenzaldehyde **6**.

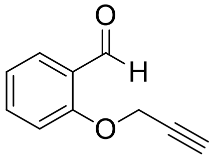

2-(Prop-2-ynyloxy)benzaldehyde (6a). General Procedure 2 was employed using 2-hydroxybenzaldehyde (**13a**) (100 mg, 0.8 mmol), K_2CO_3 (113.1 mg, 0.8 mmol) and propargyl bromide (**14**) (87.7 µl, 01.0 mmol). Purification of the crude product by flash column chromatography on silica gel afforded 111.5 mg (85%) of the indicated product. ^{1}H NMR (400 MHz, $CDCl_3$): δ 10.48 (s, 1H), 7.85 (dd, J = 8.0, 1.6 Hz, 1H), 7.57 (td, J = 7.2, 1.2 Hz, 1H), 7.10 (m, 2H), 4.83 (d, J = 2.4 Hz, 2H), 2.58 (t, J = 2.4 Hz, 1H); ^{13}C NMR (100 MHz, $CDCl_3$): δ 189.7, 159.9, 135.5, 128.7, 125.7, 121.8, 113.4, 77.8, 76.7, 56.6; IR (neat): 3268, 3873, 2116, 1679, 1595, 1480, 1456, 1398, 1286, 1263, 1192, 1104, 1044, 1006, 925, 832, 755, 675, 610, 526, 461, 441 cm^{-1}. The spectral data are in agreement with those reported previously for this compound.[5-12]

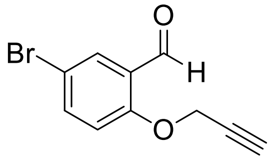

5-Bromo-2-(prop-2-ynyloxy)benzaldehyde (6b). General Procedure 2 was employed using 5-bromo-2-hydroxybenzaldehyde (**13b**) (100 mg, 0.5 mmol), K_2CO_3 (68.7 mg, 0.5 mmol) and propargyl bromide (**14**) (53.5 µl, 0.6 mmol). Purification of the crude product by flash column chromatography on silica gel afforded 101.2 mg (85%) of the indicated product. ^{1}H NMR (400 MHz, $CDCl_3$): δ 10.40 (s, 1H), 7.95 (m, 1H), 7.65 (m, 1H), 7.04 (d, J = 8.8 Hz, 1H), 4.83 (d, J = 2.4 Hz, 2H), 2.60 (t, J = 2.4 Hz, 1H); ^{13}C NMR (100 MHz, $CDCl_3$): δ 188.2, 158.8, 138.3, 131.4, 127.0, 115.6, 114.8, 77.4, 77.2, 56.9; IR (neat): 3283, 2865, 1681, 1588, 1471, 1394, 1274, 1215, 1182, 1123, 1009, 927, 875, 816, 783, 684, 645, 588, 532, 486, 440 cm^{-1}. The spectral data are in agreement with those reported previously for this compound.[7-9]

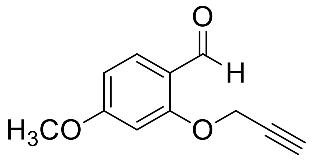

4-Methoxy-2-(prop-2-ynyloxy)benzaldehyde (6c). General Procedure 2 was employed using 2-hydroxy-4-methoxybenzaldehyde (**13c**) (90) (100 mg, 0.7 mmol), K_2CO_3 (91.1 mg, 0.7 mmol) and propargyl bromide (**14**) (70.4 μl, 0.8 mmol). Purification of the crude product by flash column chromatography on silica gel using 19:1 hexane/ethyl acetate as the eluent afforded 100.3 mg (80%) of the indicated product. ^1H NMR (400 MHz, $CDCl_3$): δ 10.31 (s, 1H), 7.84 (d, *J* = 9.2 Hz, 1H), 6.60 (m, 2H), 4.81 (d, *J* = 2.4 Hz, 2H), 3.89 (s, 3H), 2.59 (t, *J* = 2.4 Hz, 1H) ; ^{13}C NMR (100 MHz, $CDCl_3$): δ 188.3, 166.1, 161.7, 130.9, 119.8, 107.1, 99.7, 77.8, 76.8, 56.6, 55.9; IR (neat): 3228, 2123, 1665, 1600, 1504, 1444, 1398, 1371, 1314, 1293, 1264, 1196, 1168, 1106, 1048, 1008, 938, 850, 821, 782, 750, 691, 636, 603, 561, 463, 425 cm^{-1}. The spectral data are in agreement with those reported previously for this compound.[10,12]

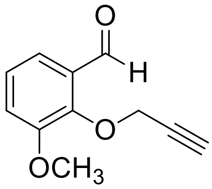

3-Methoxy-2-(prop-2-ynyloxy)benzaldehyde (6d). General Procedure 2 was employed using 2-hydroxy-3-methoxybenzaldehyde (**13d**) (91) (100 mg, 0.7 mmol), K_2CO_3 (91.1 mg, 0.7 mmol) and propargyl bromide (**14**) (70.4 μl, 0.8 mmol). Purification of the crude product by flash column chromatography on silica gel afforded 101.6 mg (81%) of the indicated product. ^1H NMR (400 MHz, $CDCl_3$): δ 10.51 (s, 1H), 7.47 (dd, *J* = 6.4, 2.0 Hz, 1H), 7.19 (m, 2H), 4.90 (d, *J* = 2.4 Hz, 2H), 3.92 (s, 3H), 2.49 (t, *J* = 2.4 Hz, 1H); ^{13}C NMR (100 MHz, $CDCl_3$): δ 190.8, 153.1, 149.7, 131.4, 125.1, 119.1, 118.0, 78.5, 77.1, 61.1, 56.3; IR (neat): 3266, 2939, 2889, 1682, 1583, 1478, 1437, 1383, 1248, 1202, 1178, 1066, 981, 910, 782, 749, 649, 529 cm^{-1}. The spectral data are in agreement with those reported previously for this compound.[7,10-13]

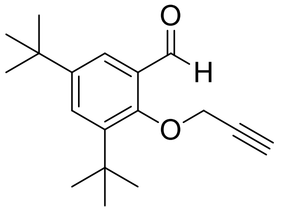

3,5-Di-*tert*-butyl-2-(prop-2-ynyloxy)benzaldehyde (6e). General Procedure 2 was employed using 3,5-di-*tert*-butyl-2-hydroxybenzaldehyde (**13e**) (88) (100 mg, 0.4 mmol), K_2CO_3 (59.0 mg, 0.4 mmol) and propargyl bromide (**14**) (46.0 μl, 0.5 mmol). Purification of the crude product by flash column chromatography on silica gel afforded 97.1 mg (83%) of the indicated product. ^1H NMR (400 MHz, $CDCl_3$): δ 10.38 (s, 1H), 7.72 (d, *J* = 2.4 Hz, 1H), 7.65 (d, *J* = 2.8 Hz, 1H), 4.64 (d, *J* = 2.4 Hz, 2H), 2.62 (t, *J* = 2.4 Hz, 1H), 1.46 (s, 9H), 1.33 (s, 9H); ^{13}C NMR (100 MHz, $CDCl_3$): δ 191.0, 158.6, 147.3, 143.5, 131.2, 129.8, 124.8, 78.4, 76.9, 65.2, 35.6, 35.0, 31.5, 31.2; IR (neat): 3287, 2958, 1738, 1689, 1593, 1441, 1361, 1234, 1199, 1161, 1113, 989, 961, 892, 814, 750, 686, 647, 600, 548 cm^{-1}. The spectral data are in agreement with those reported previously for this compound.[7,10,11]

Copies of ¹H and ¹³C NMR Spectra

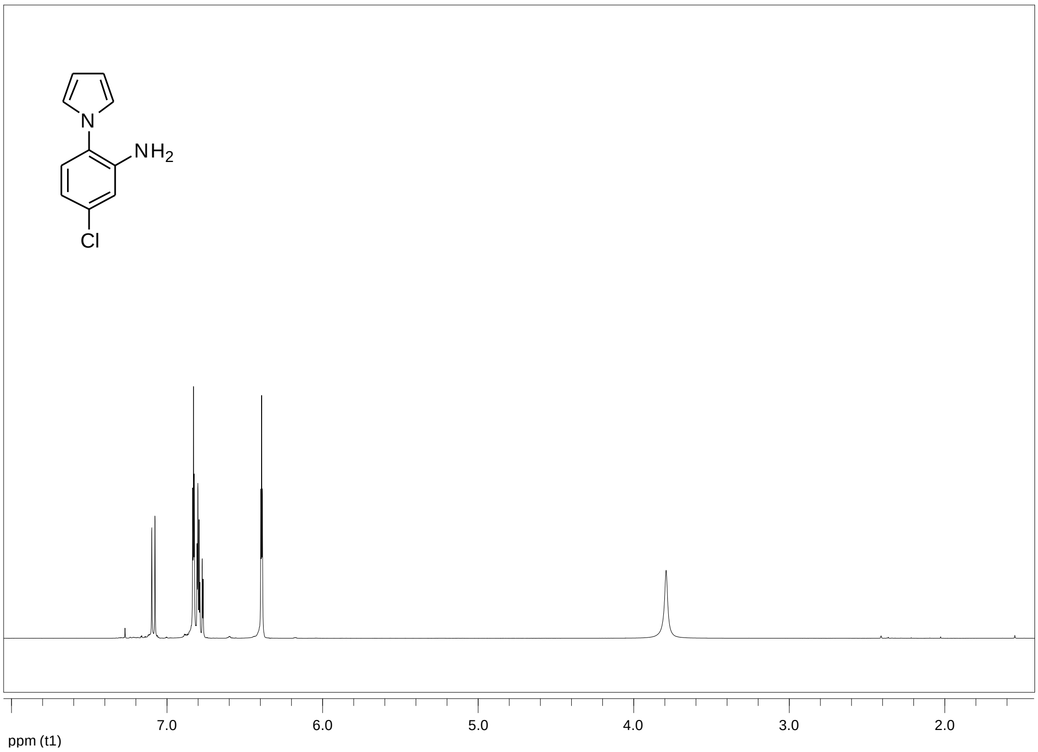

Figure S1. ^{1}H NMR Spectrum of compound **1b**.

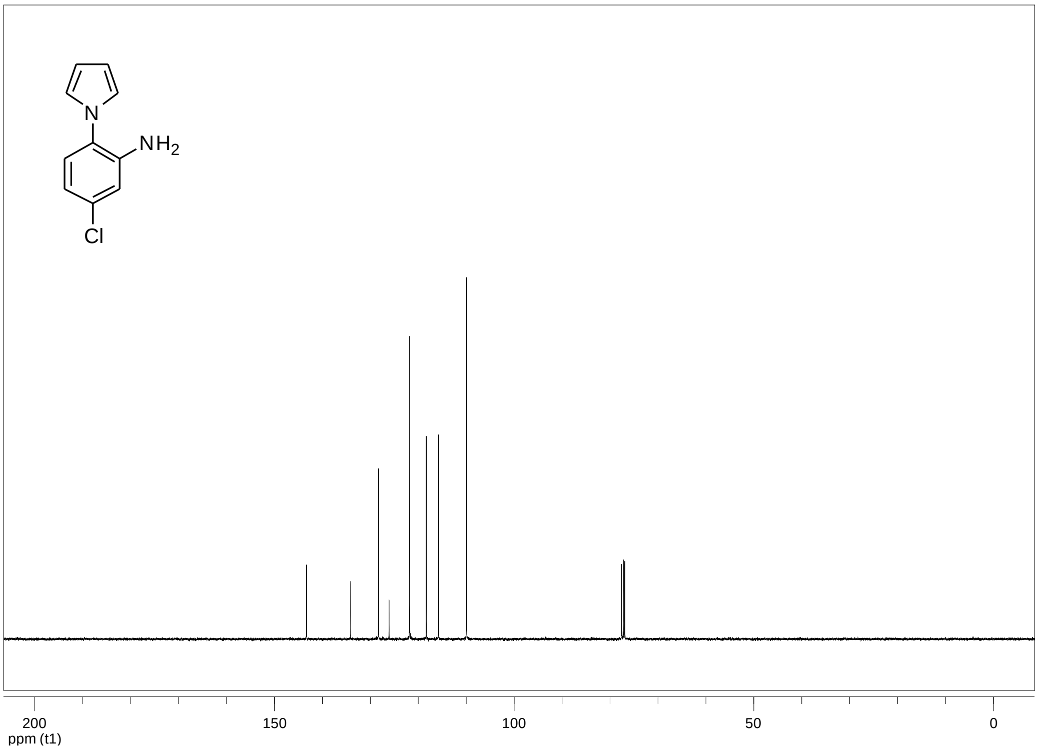

Figure S2. ^{13}C NMR Spectrum of compound **1b**.

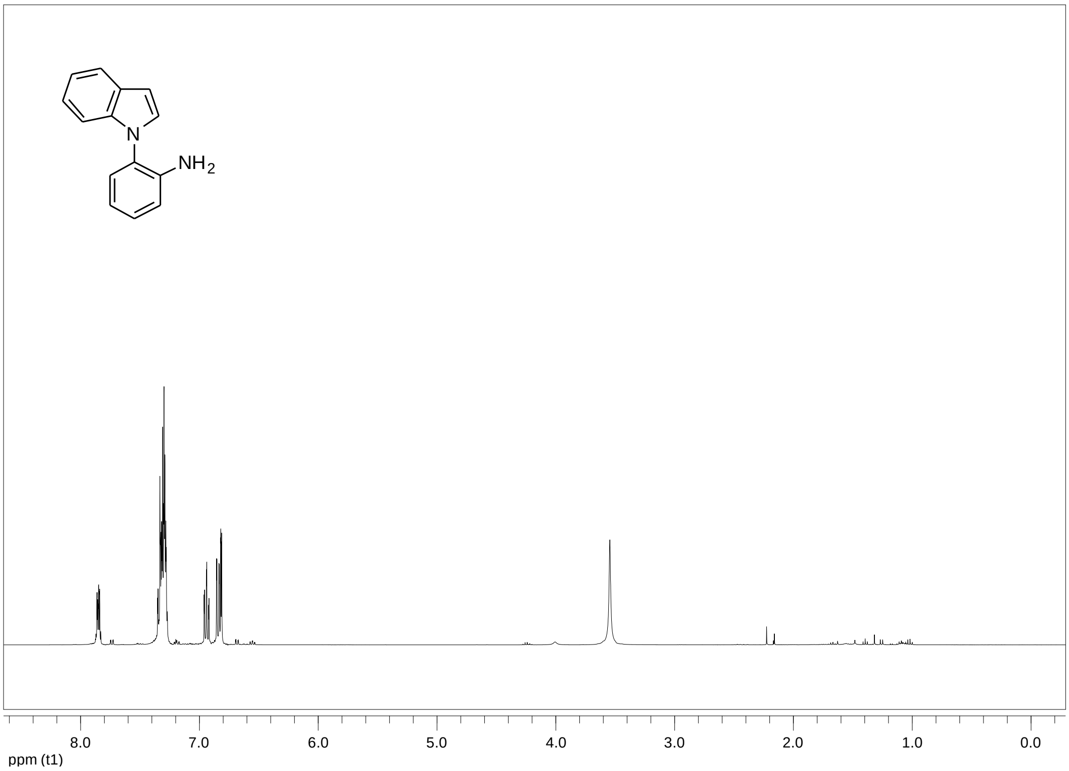

Figure S3. ^{1}H NMR Spectrum of compound **1c**.

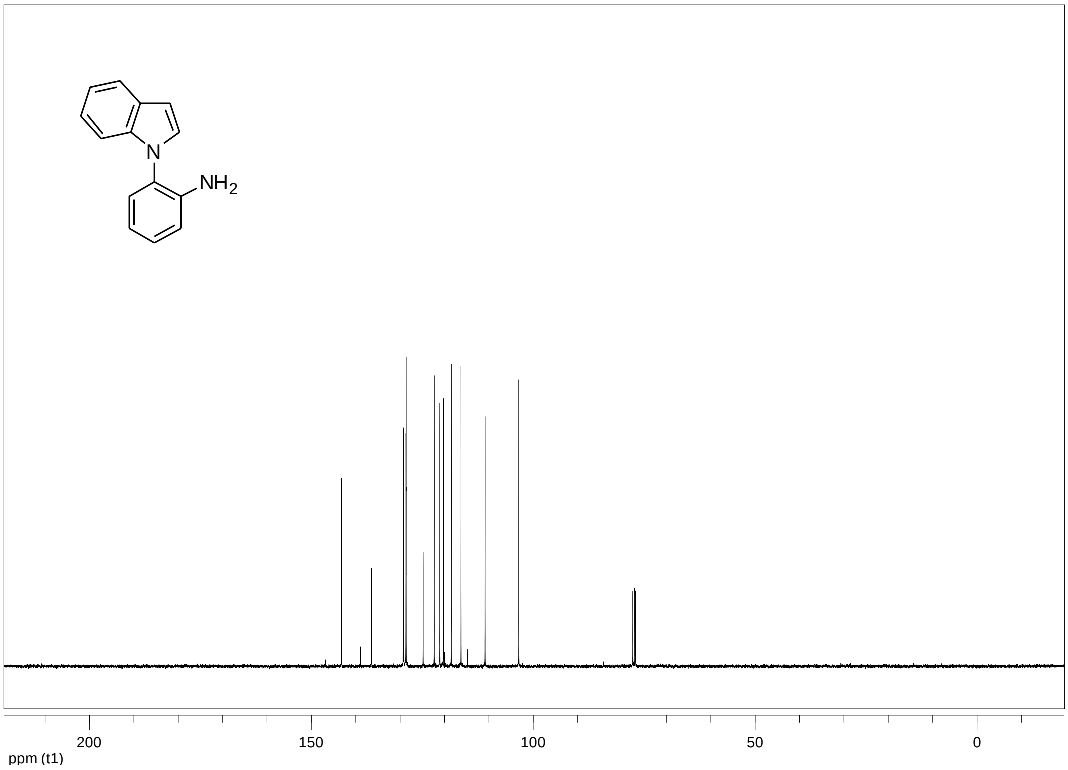

Figure S4. ^{13}C NMR Spectrum of compound **1c**.

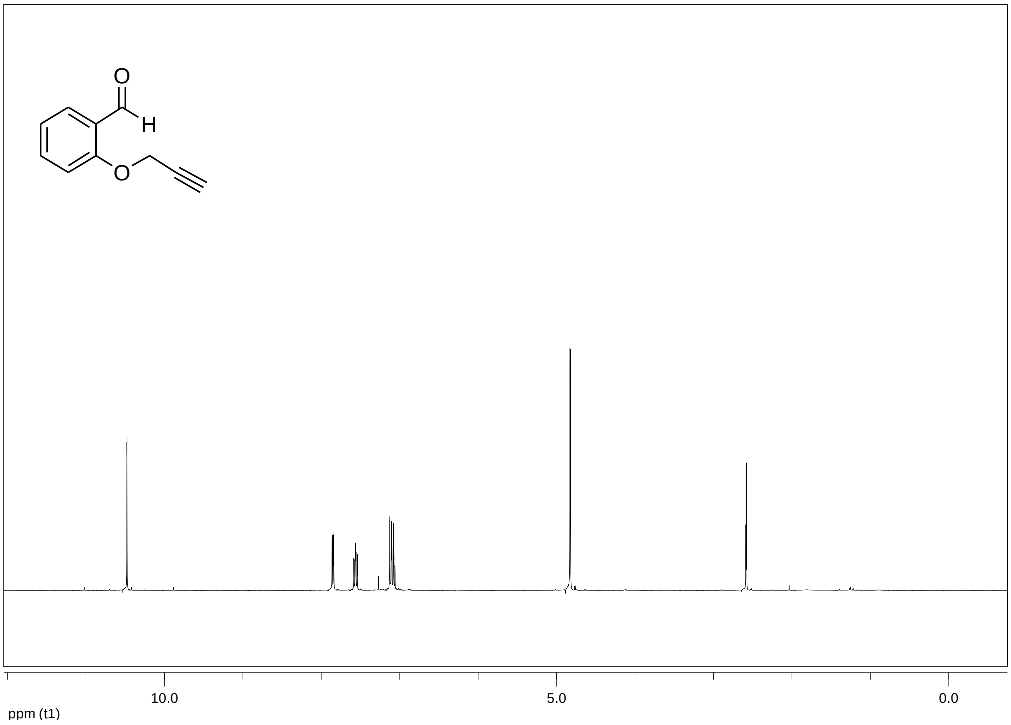

Figure S5. ^{1}H NMR Spectrum of compound **6a**.

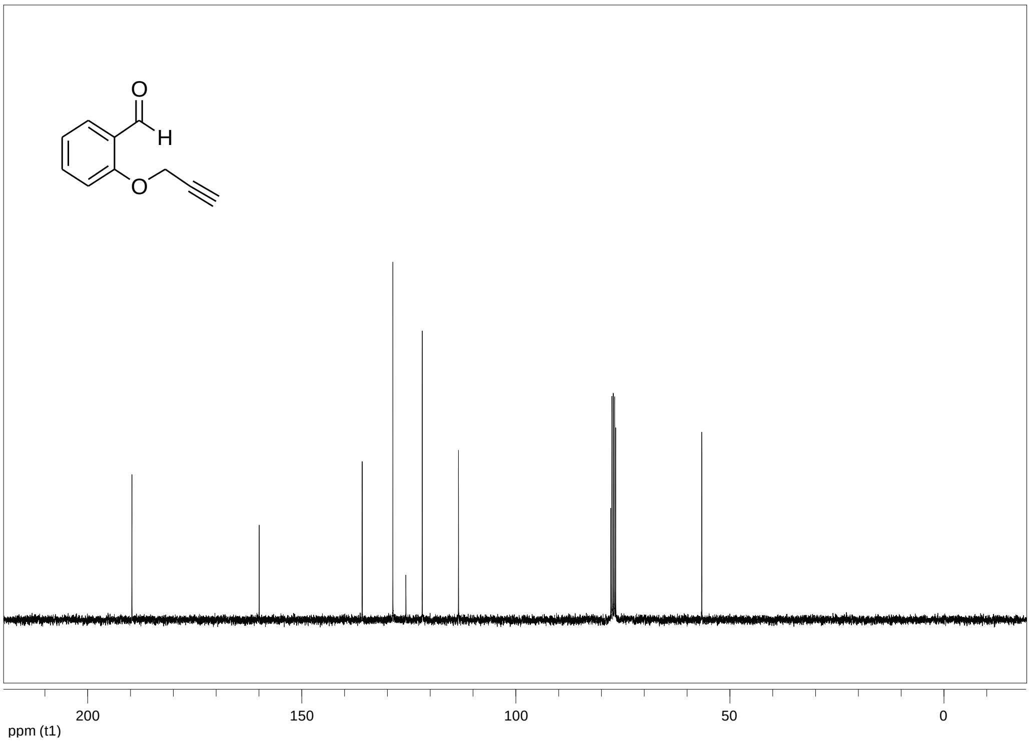

Figure S6. ^{13}C NMR Spectrum of compound **6a**.

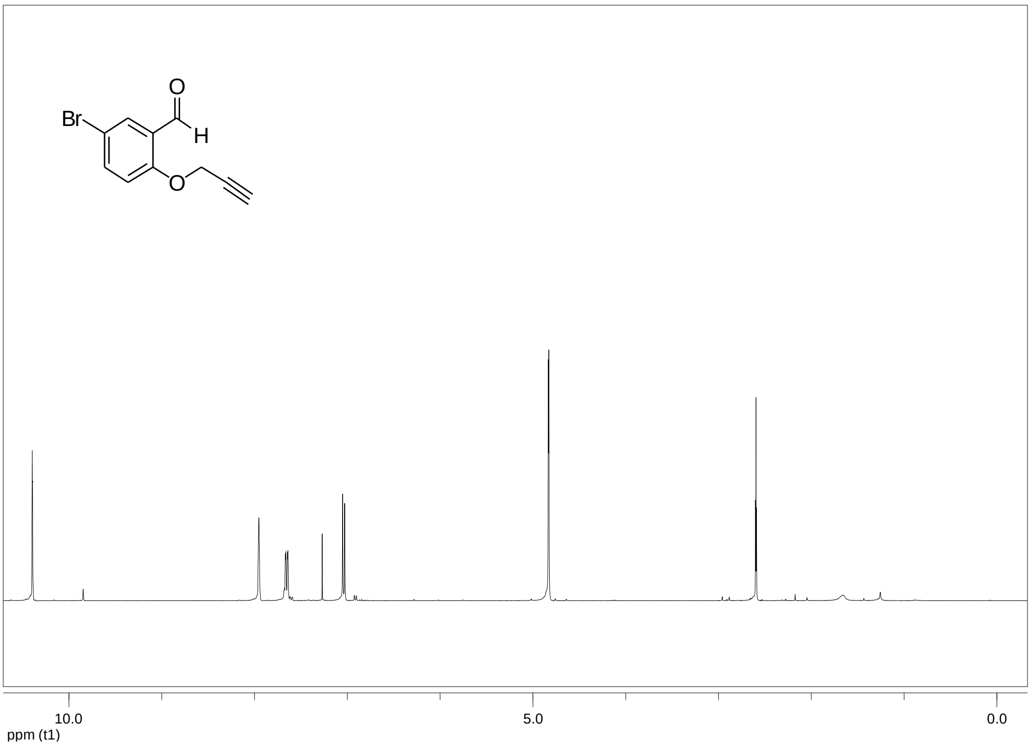

Figure S7. ^{1}H NMR Spectrum of compound **6b**.

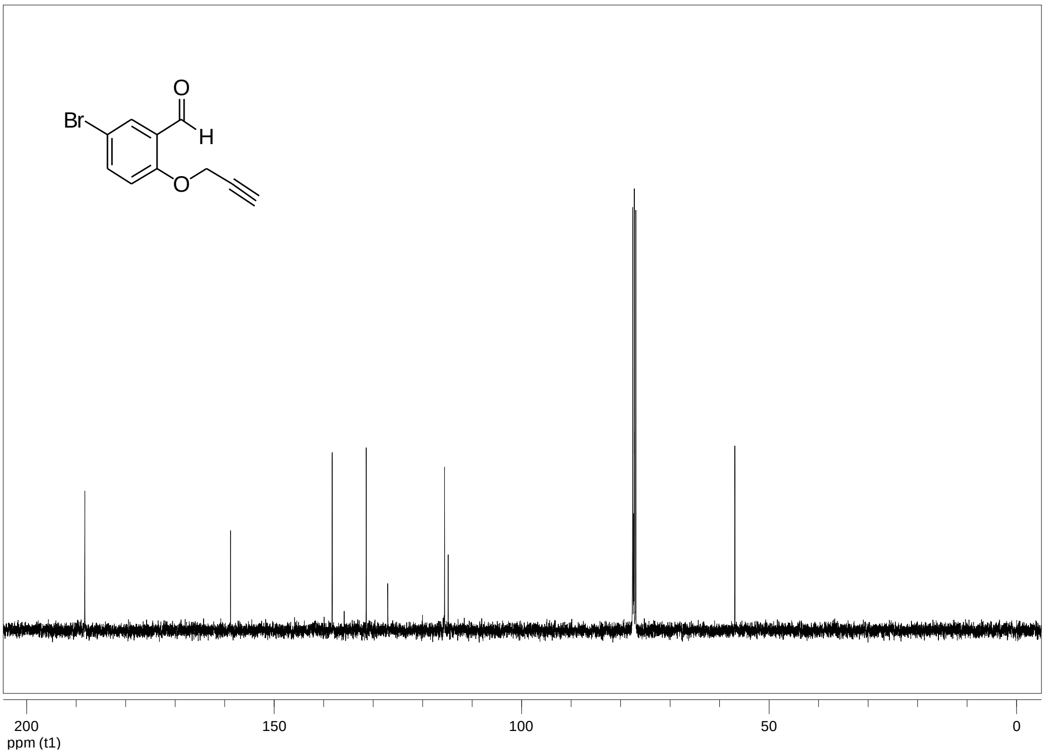

Figure S8. ^{13}C NMR Spectrum of compound **6b**.

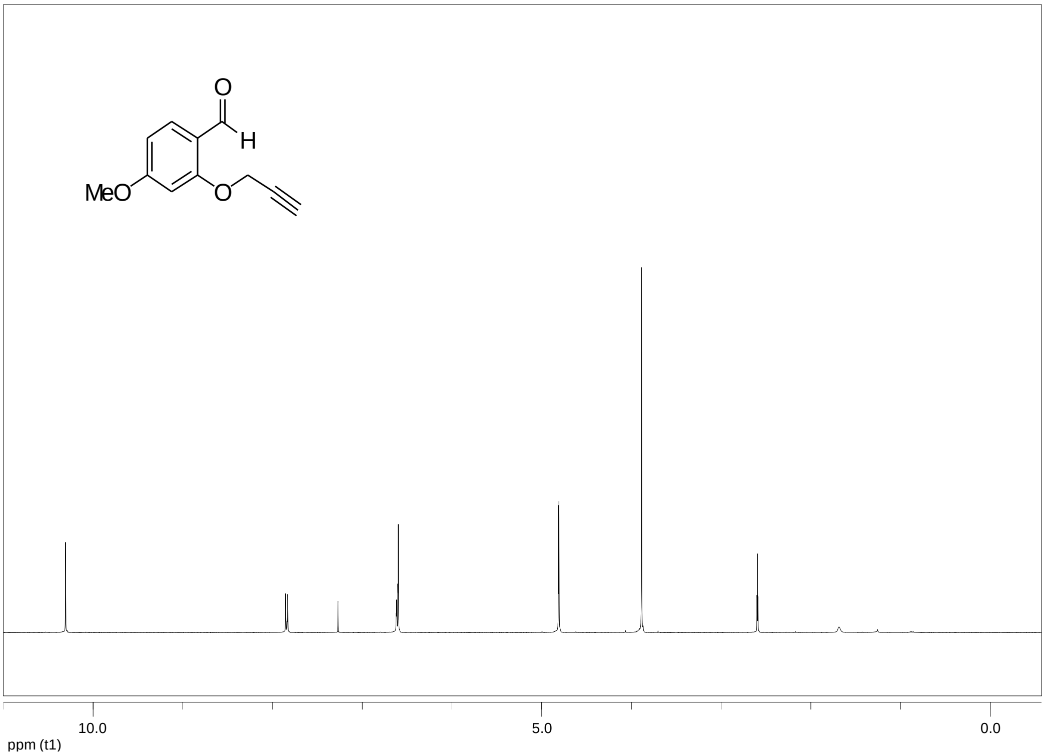

Figure S9. ^{1}H NMR Spectrum of compound **6c**.

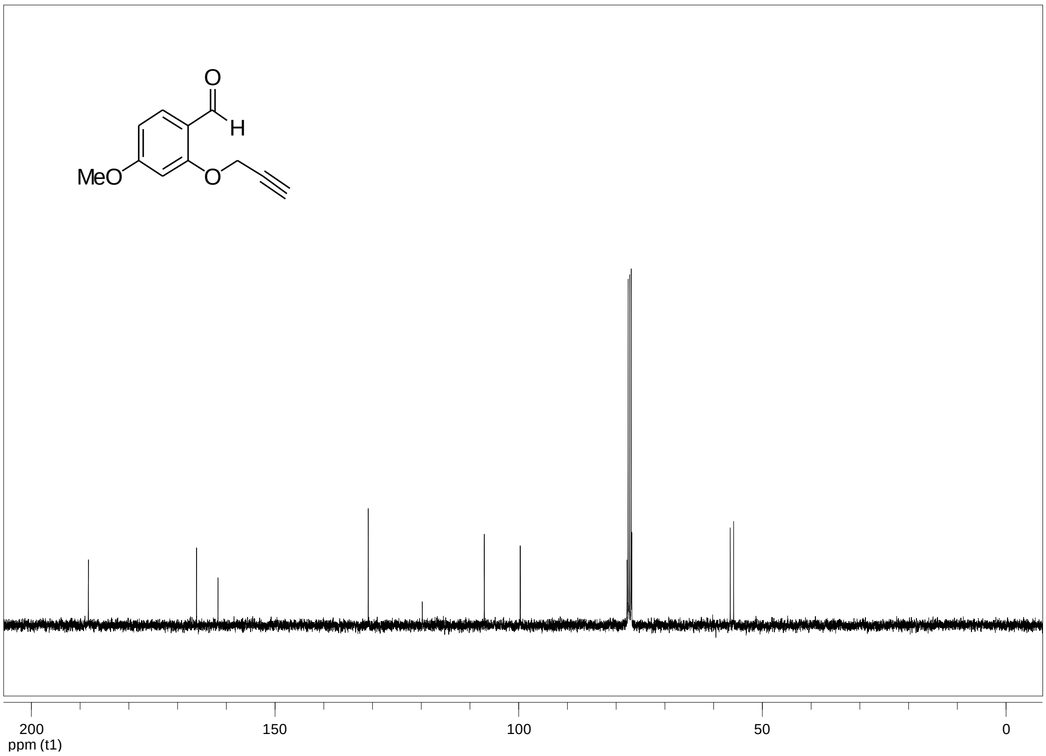

Figure S10. ^{13}C NMR Spectrum of compound **6c**.

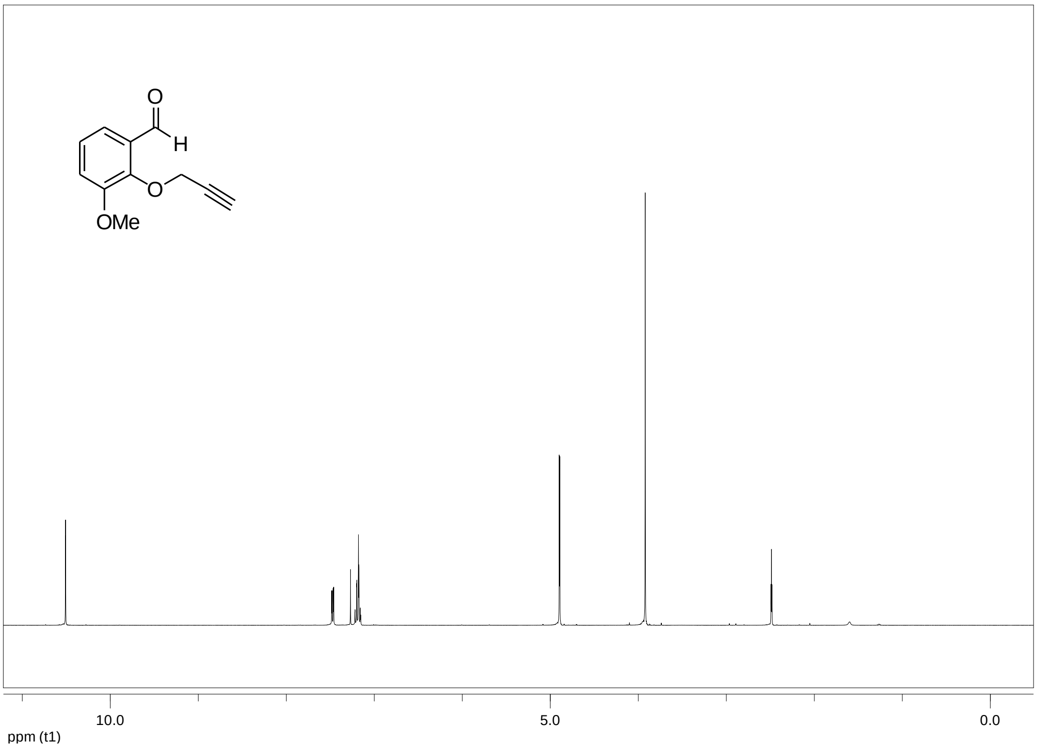

Figure S11. ^{1}H NMR Spectrum of compound **6d**.

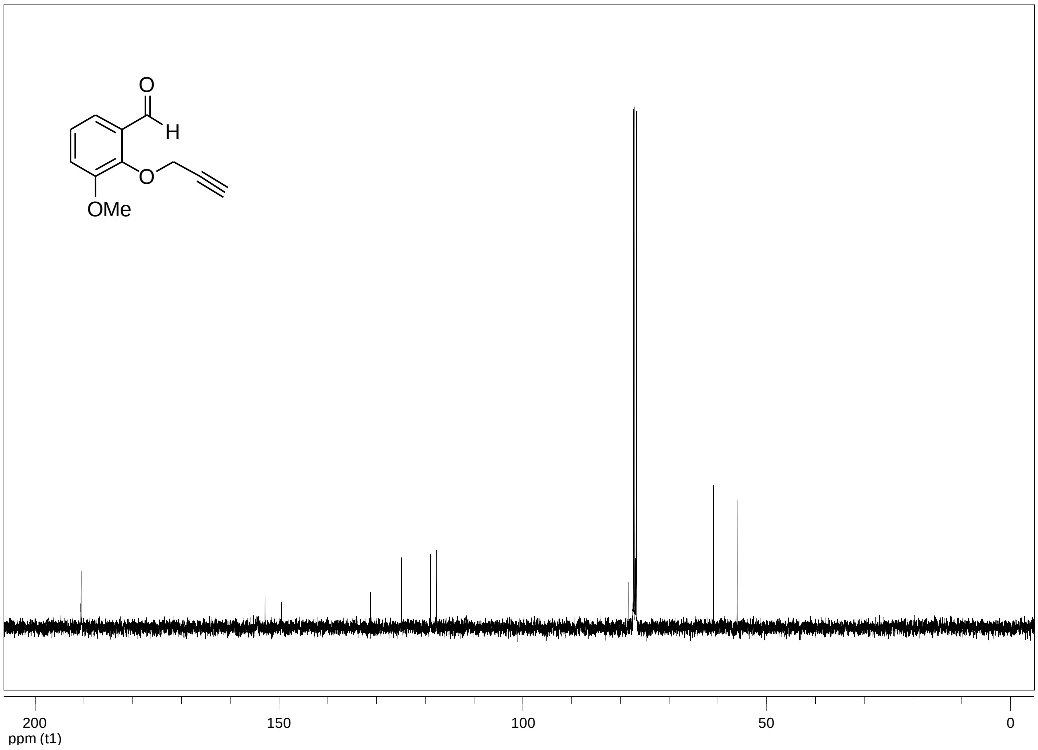

Figure S12. ^{13}C NMR Spectrum of compound **6d**.

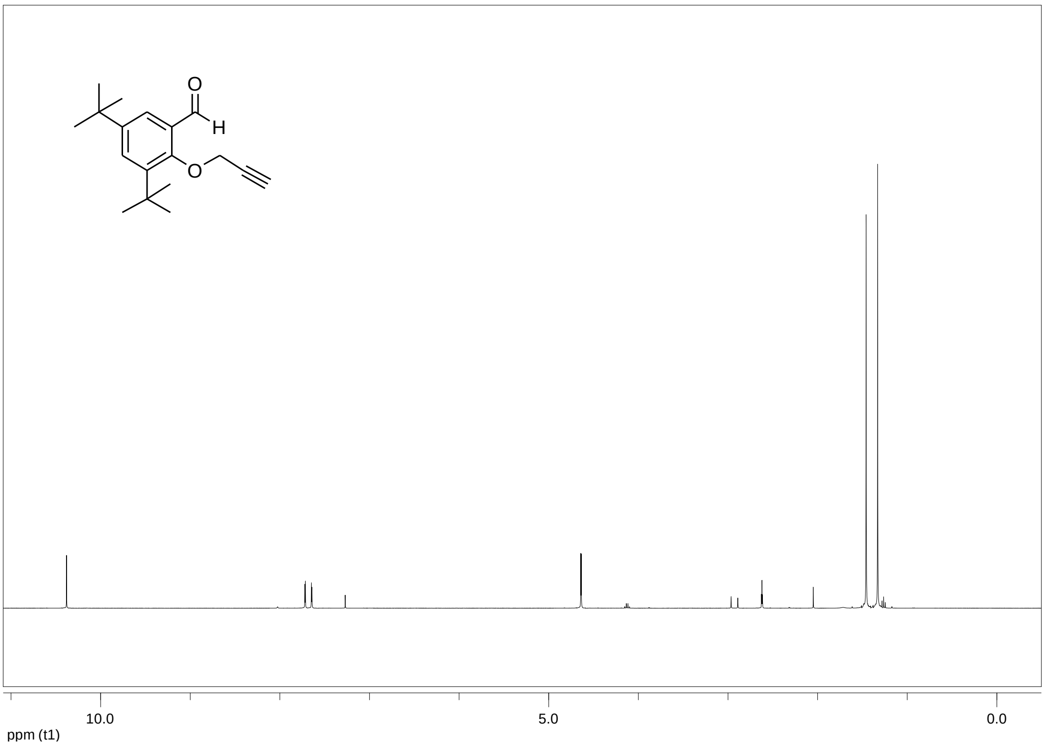

Figure S13. ^{1}H NMR Spectrum of compound **6e**.

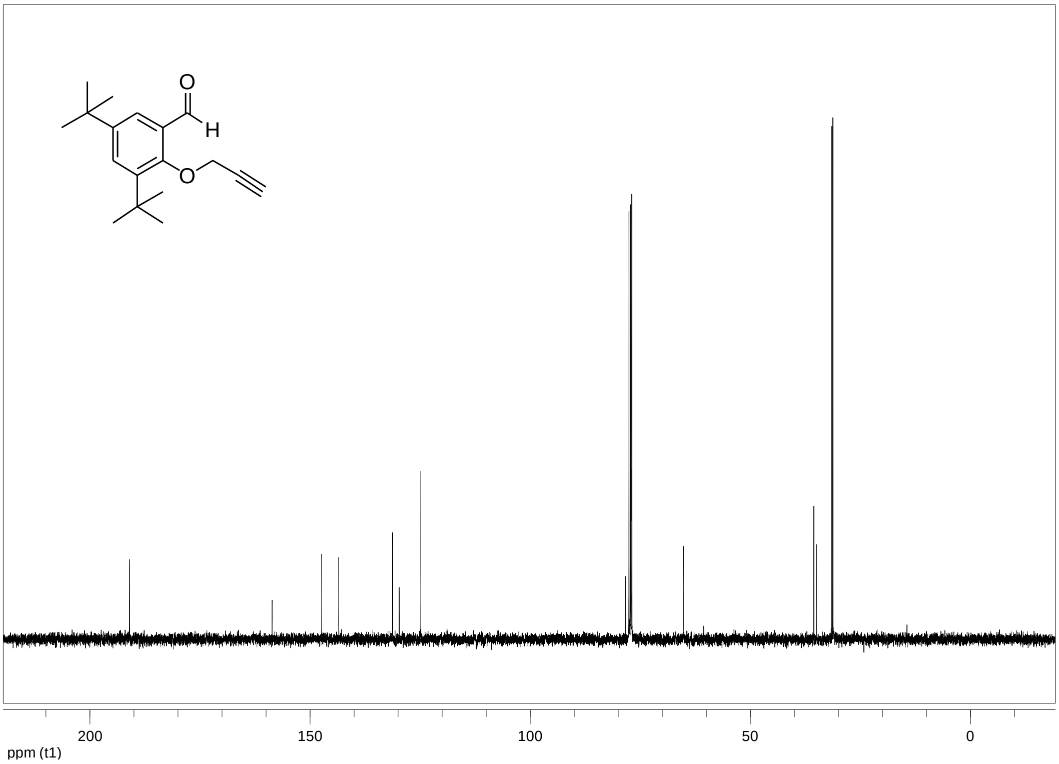

Figure S14. ^{13}C NMR Spectrum of compound **6e**.

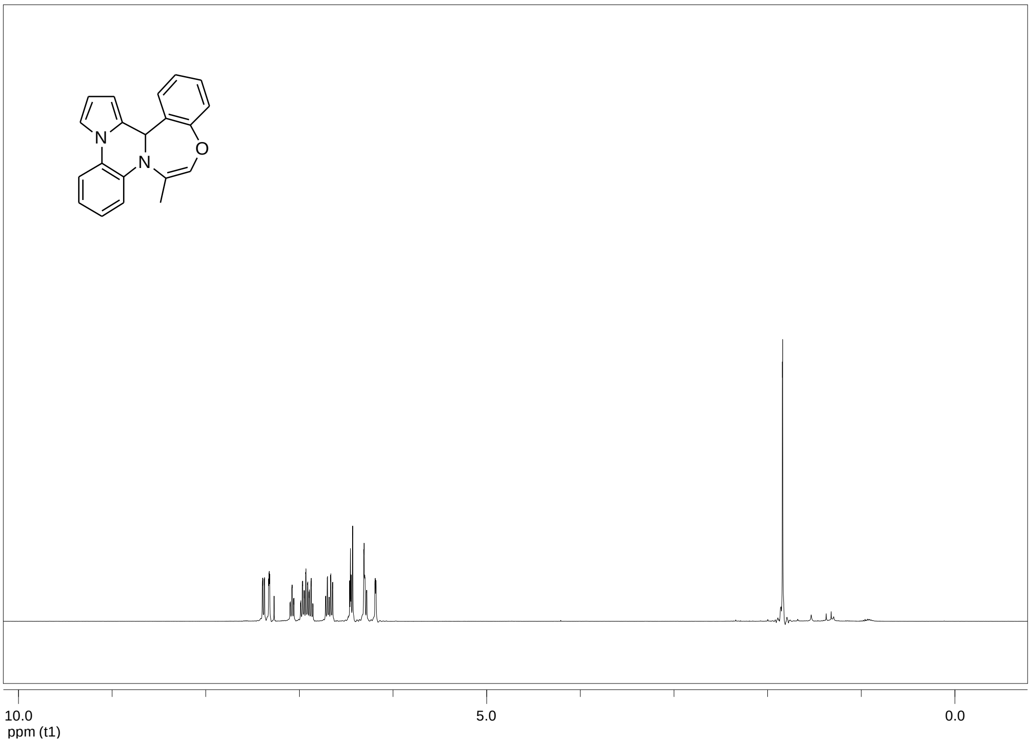

Figure S15. ^{1}H NMR Spectrum of compound **10a**.

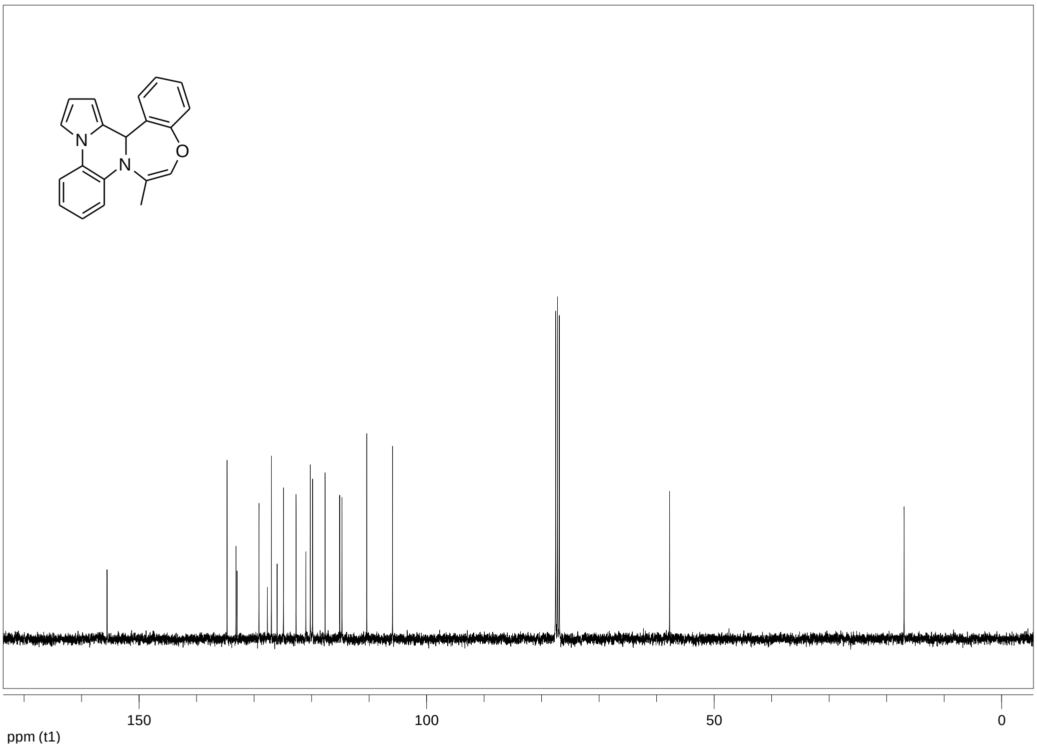

Figure S16. ^{13}C NMR Spectrum of compound **10a**.

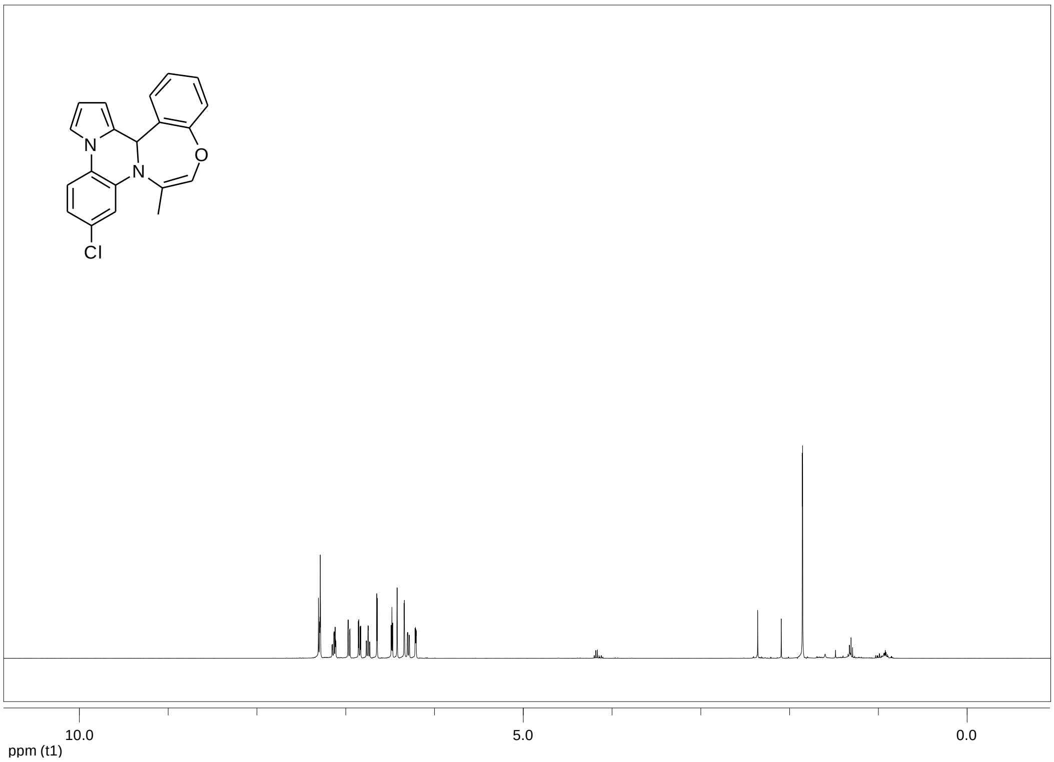

Figure S17. ^{1}H NMR Spectrum of compound **10b**.

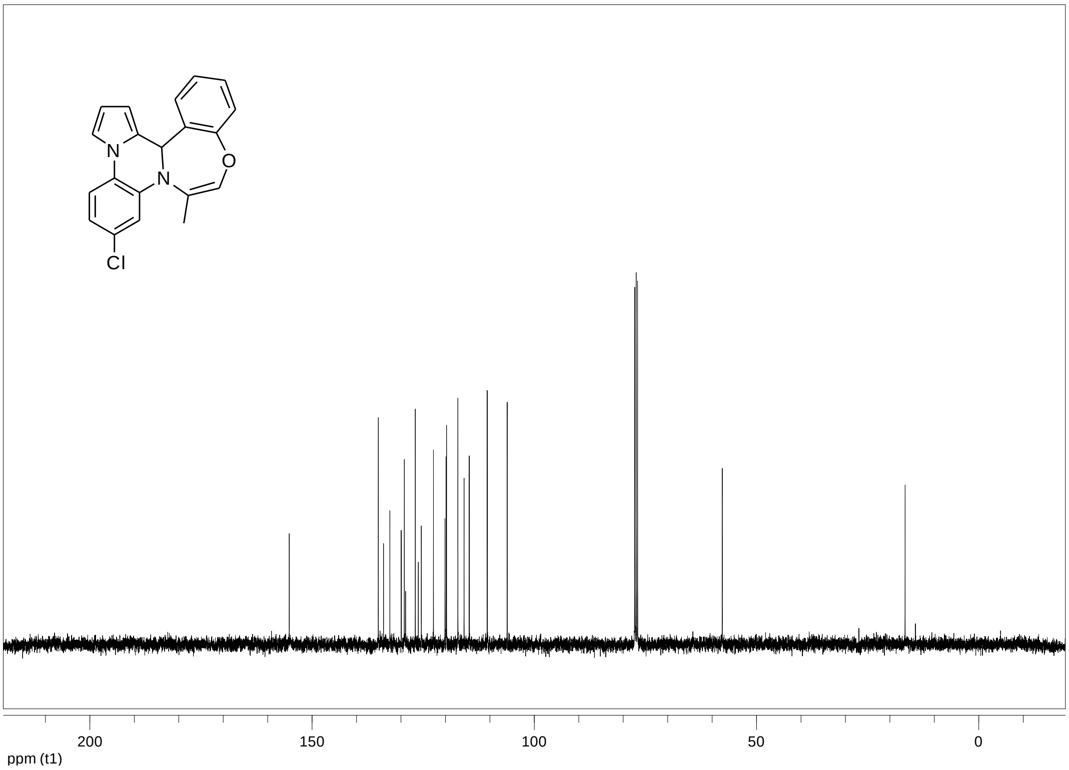

Figure S18. ^{13}C NMR Spectrum of compound **10b**.

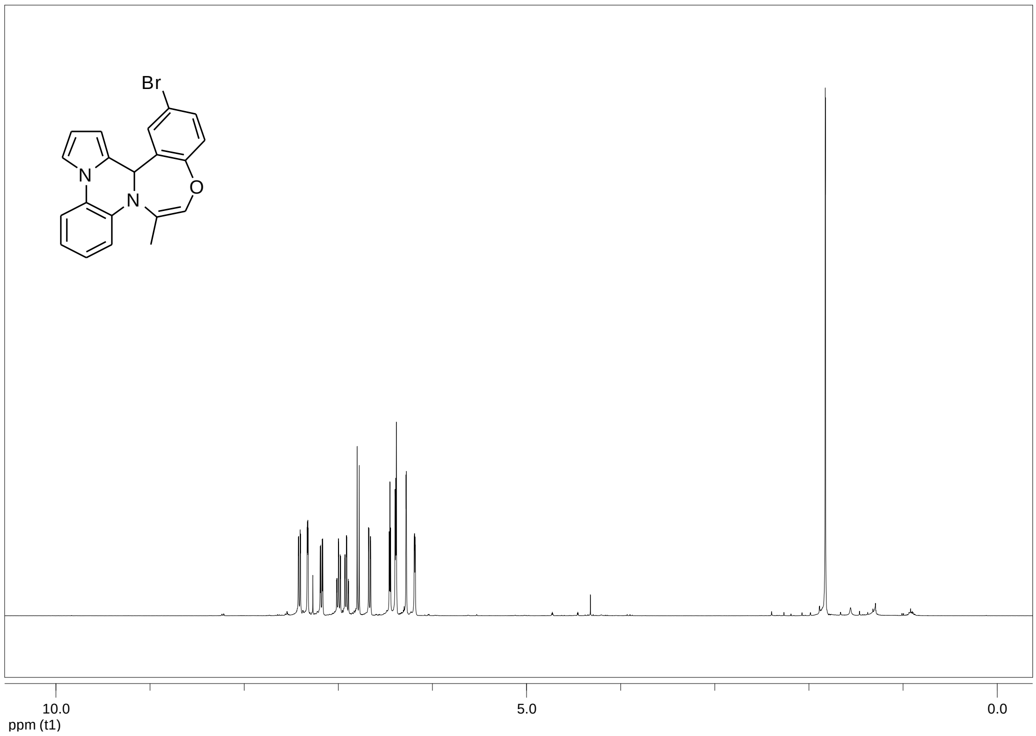

Figure S19. ^{1}H NMR Spectrum of compound **10c**.

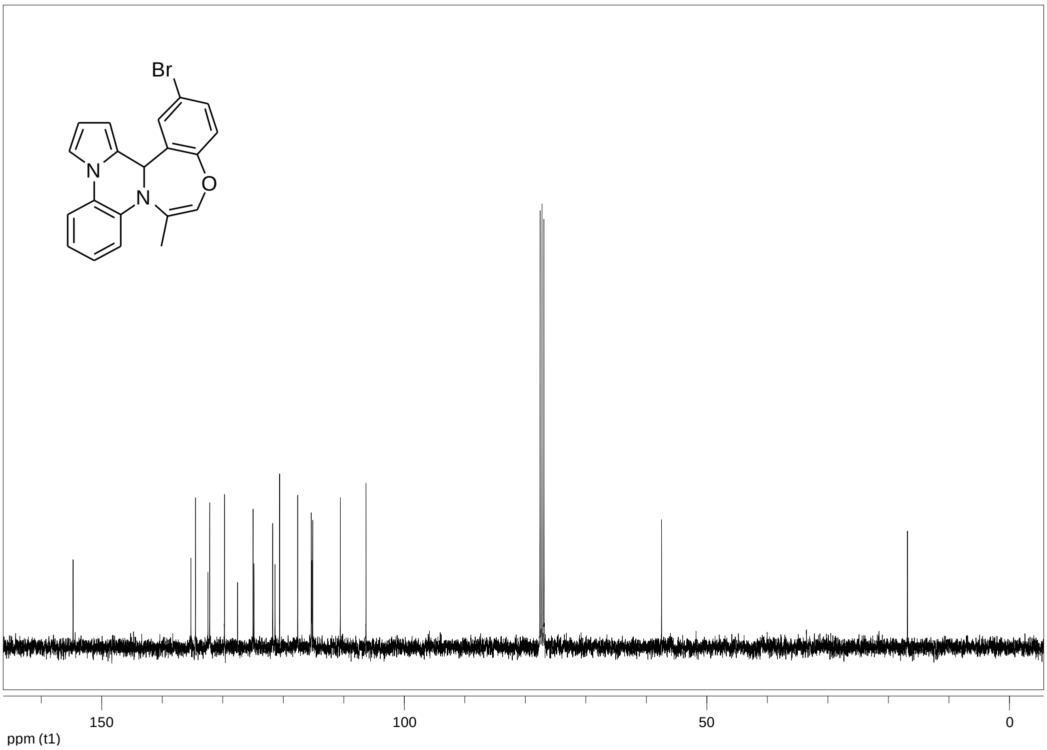

Figure S20. ^{13}C NMR Spectrum of compound **10c**.

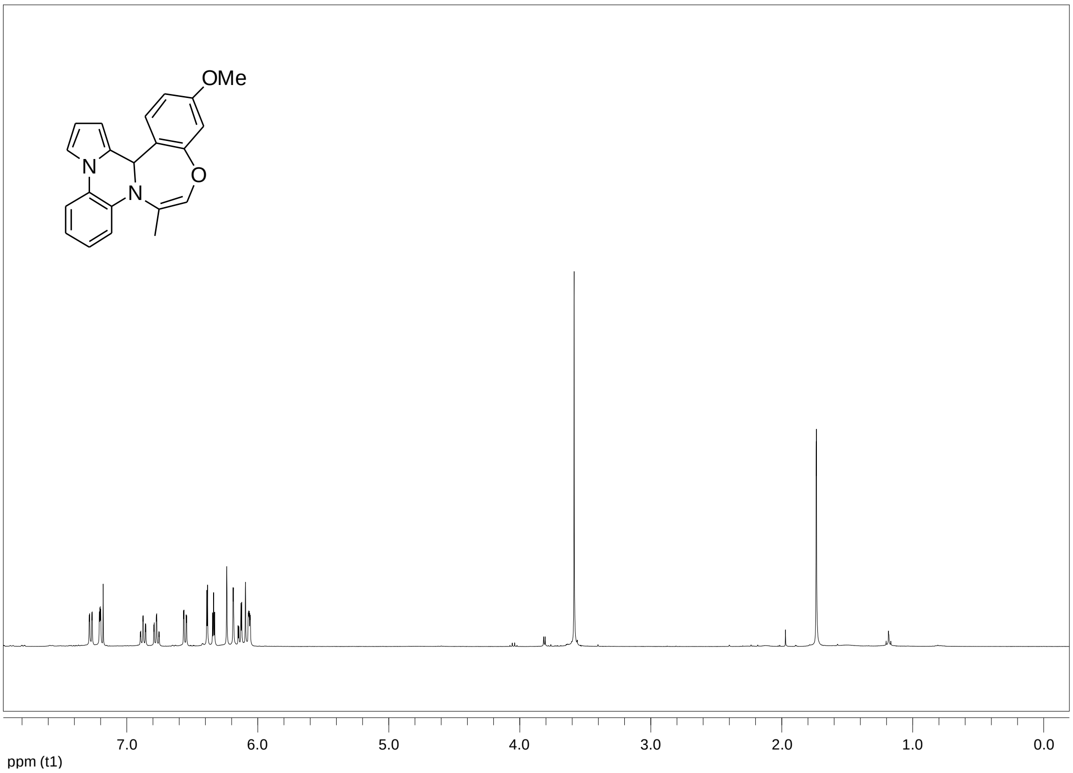

Figure S21. ^{1}H NMR Spectrum of compound **10d**.

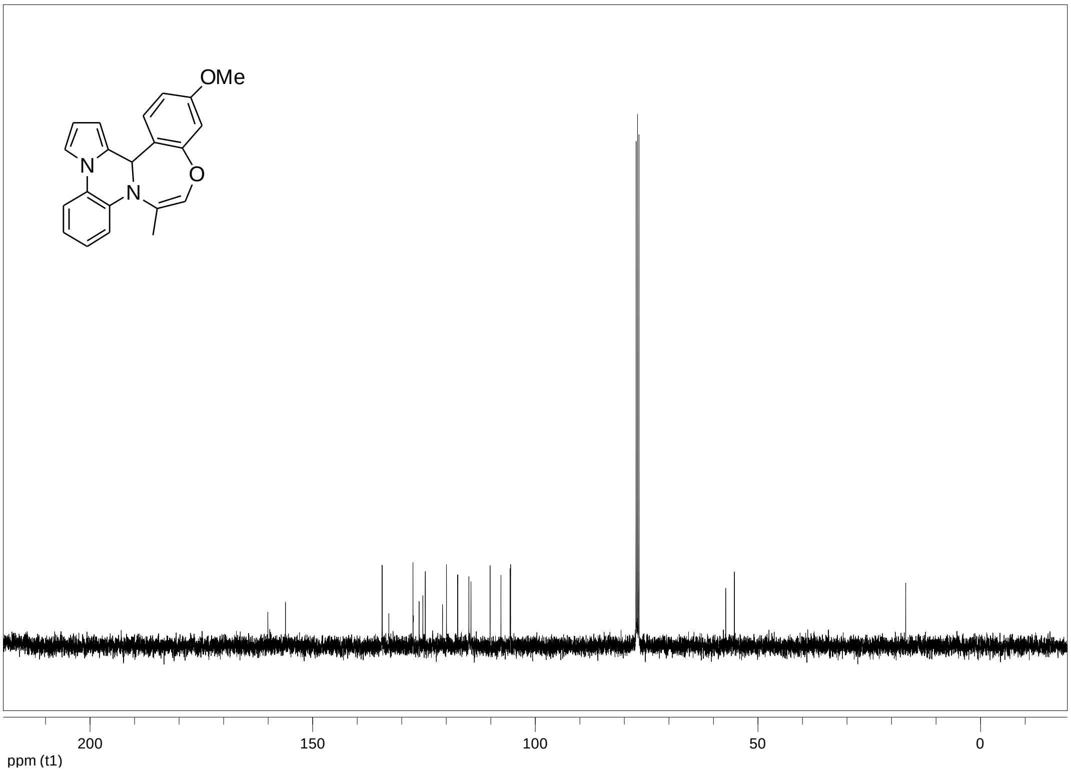

Figure S22. ^{13}C NMR Spectrum of compound **10d**.

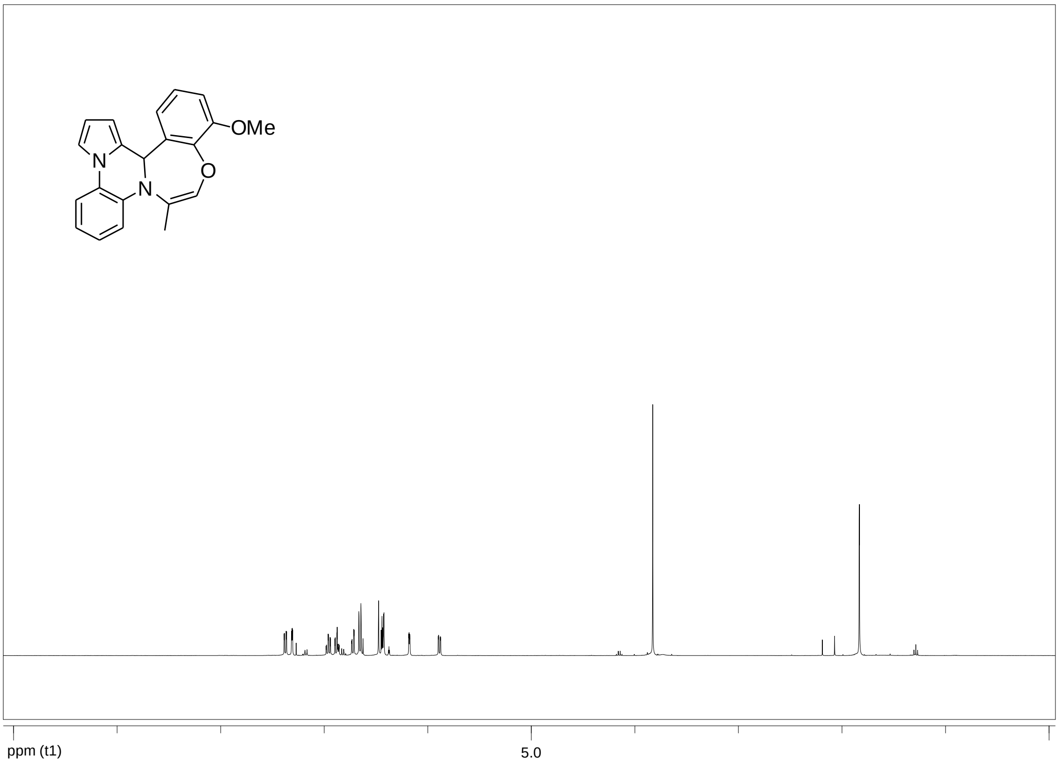

Figure S23. ^{1}H NMR Spectrum of compound **10e**.

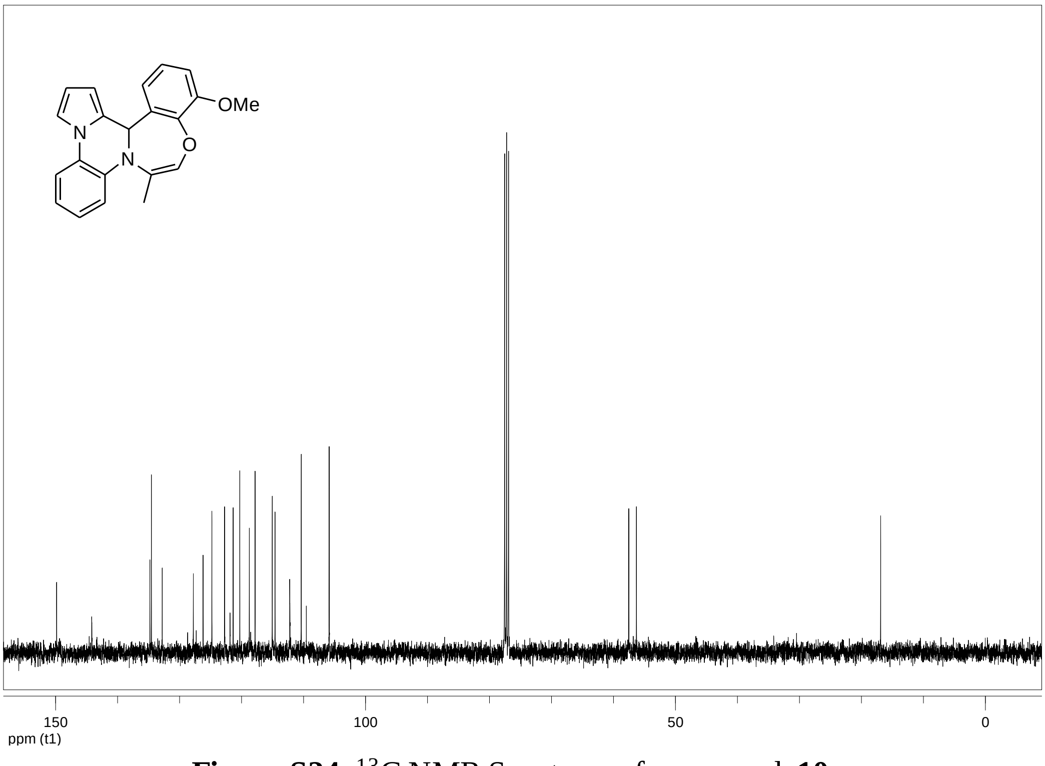

Figure S24. ^{13}C NMR Spectrum of compound **10e**.

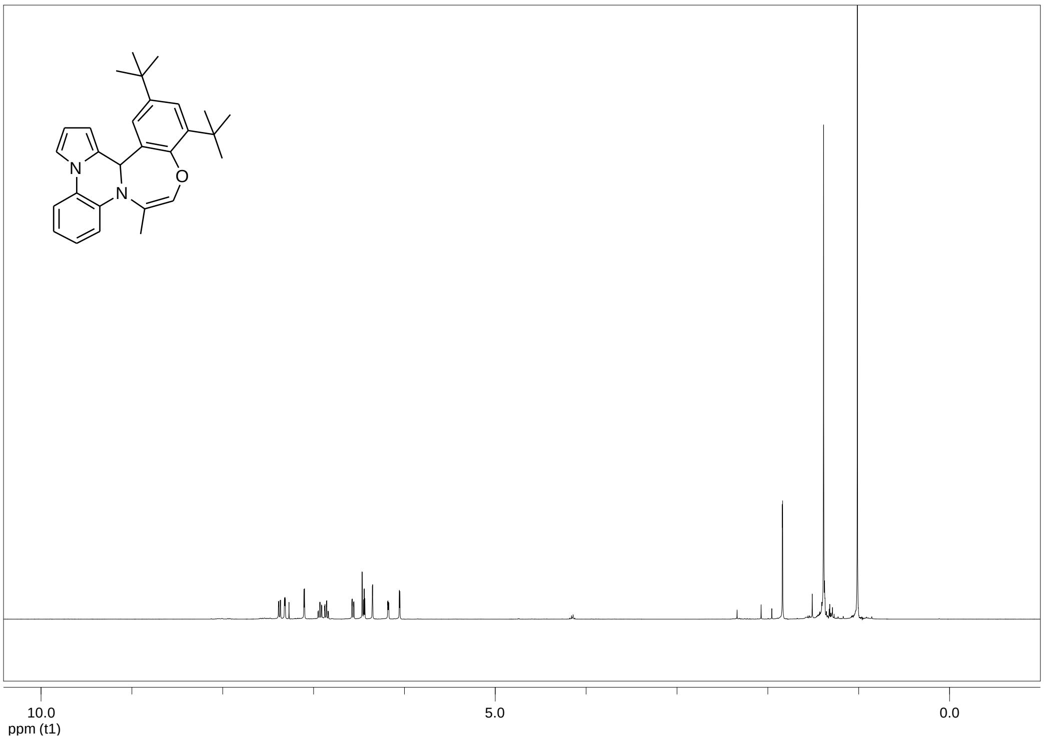

Figure S25. ^{1}H NMR Spectrum of compound **10f**.

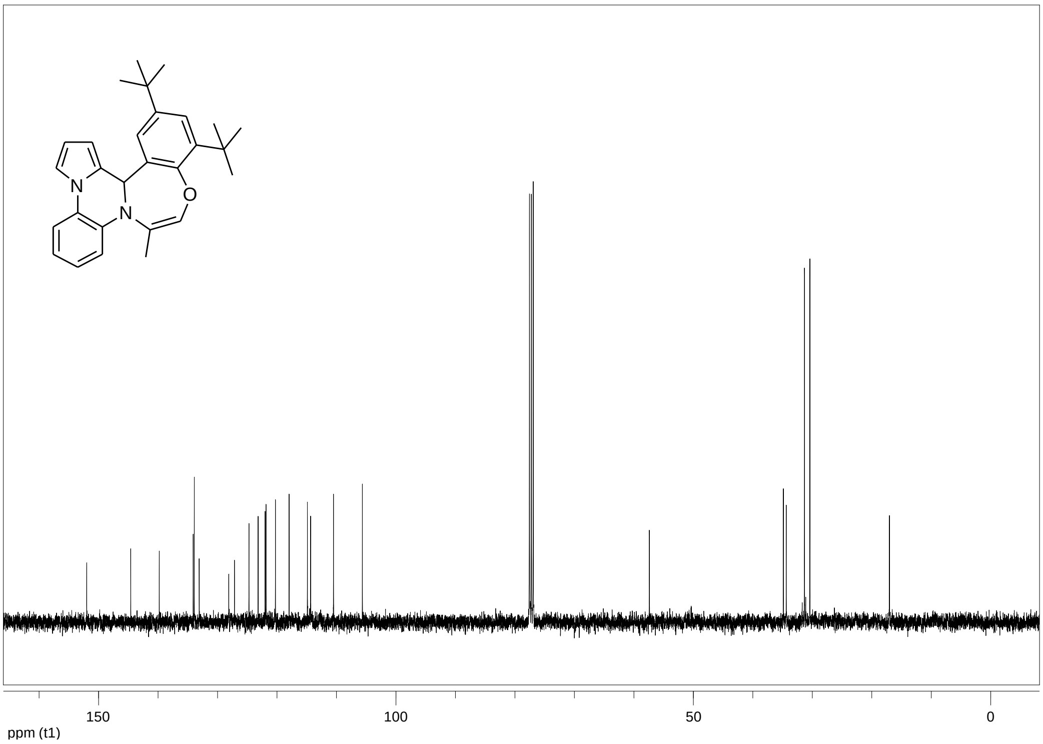

Figure S26. ^{13}C NMR Spectrum of compound **10f**.

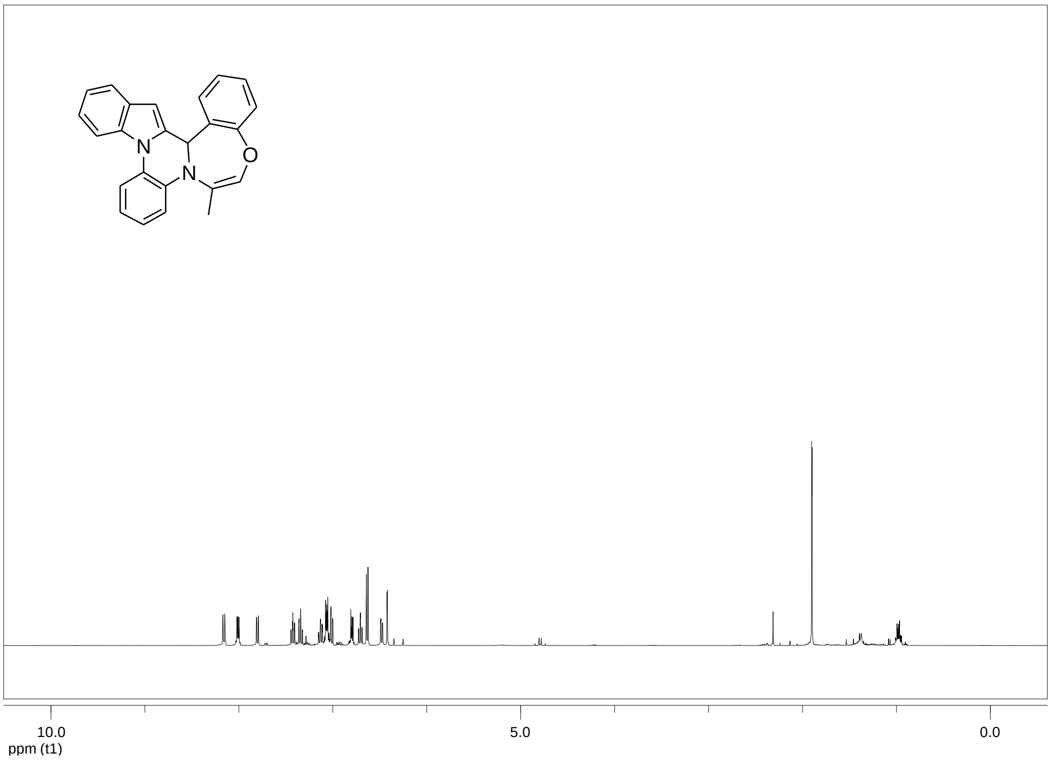

Figure S27. ^{1}H NMR Spectrum of compound **10g**.

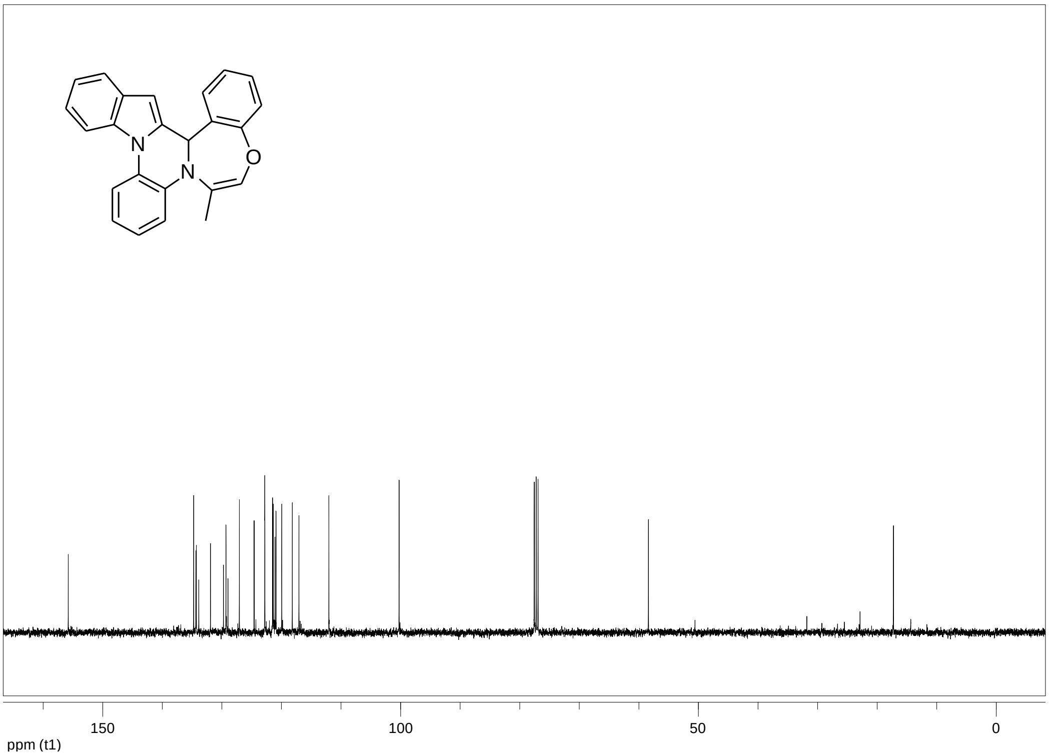

Figure S28. ^{13}C NMR Spectrum of compound **10g**.

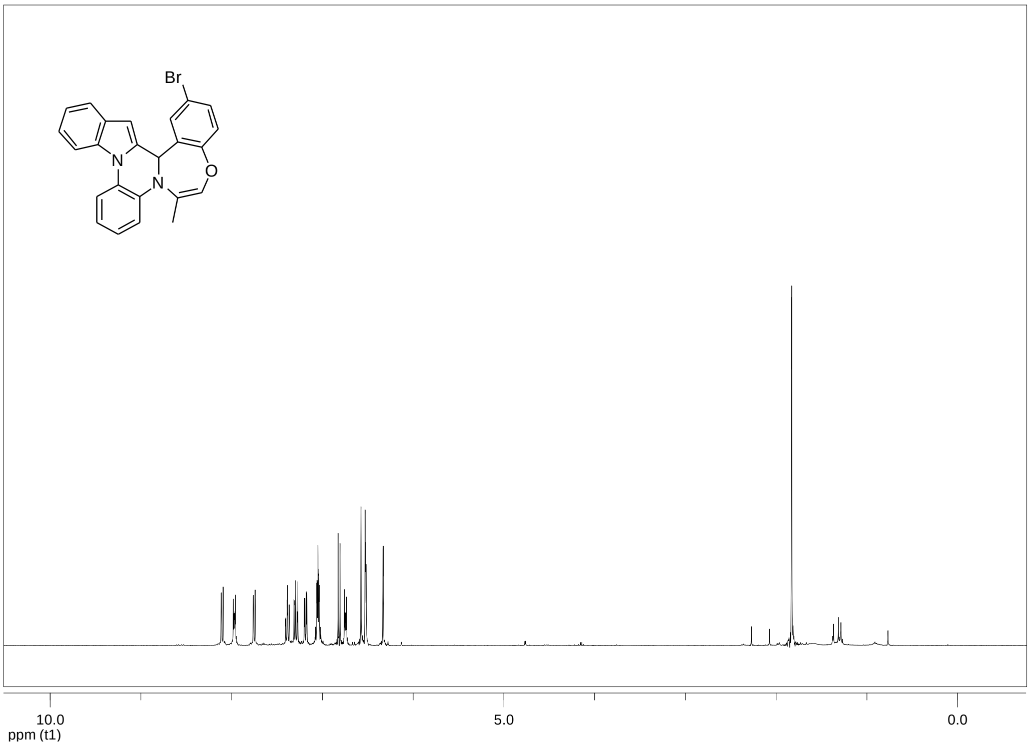

Figure S29. ^{1}H NMR Spectrum of compound **10h**.

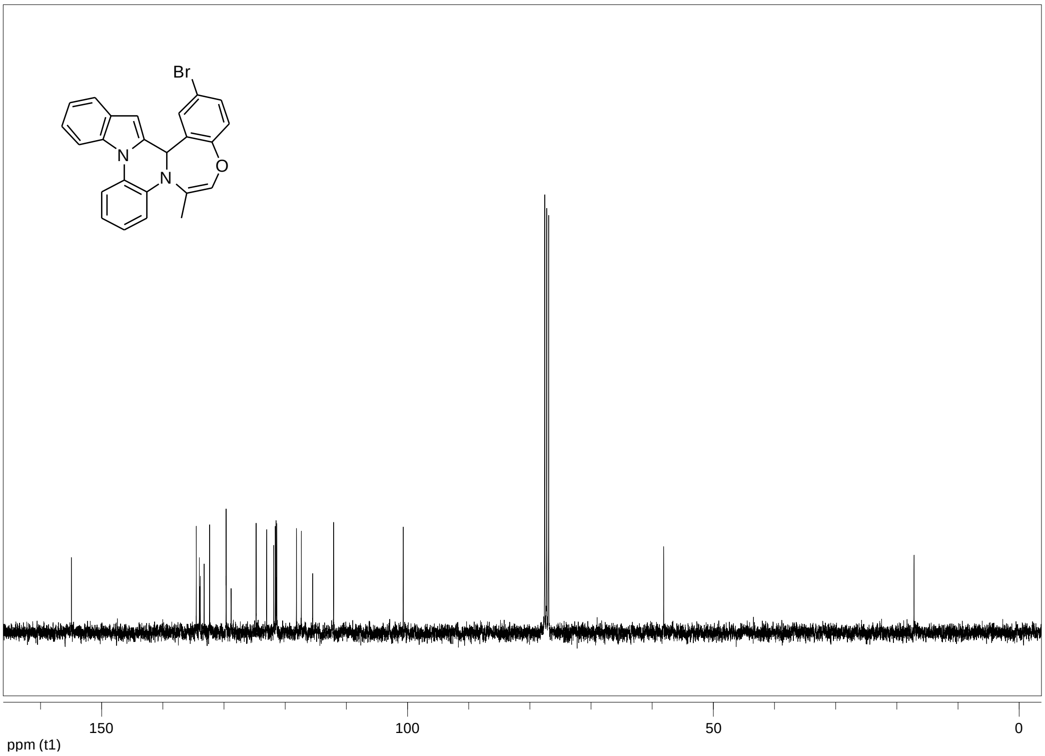

Figure S30. ^{13}C NMR Spectrum of compound **10h**.

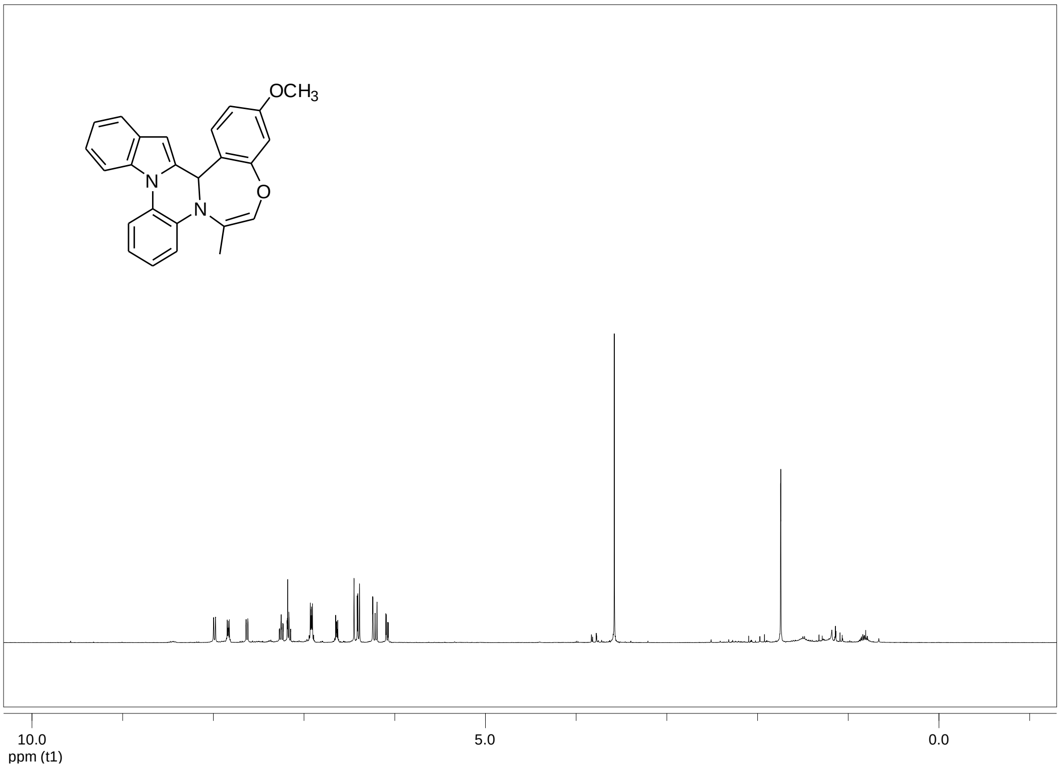

Figure S31. ^{1}H NMR Spectrum of compound **10i**.

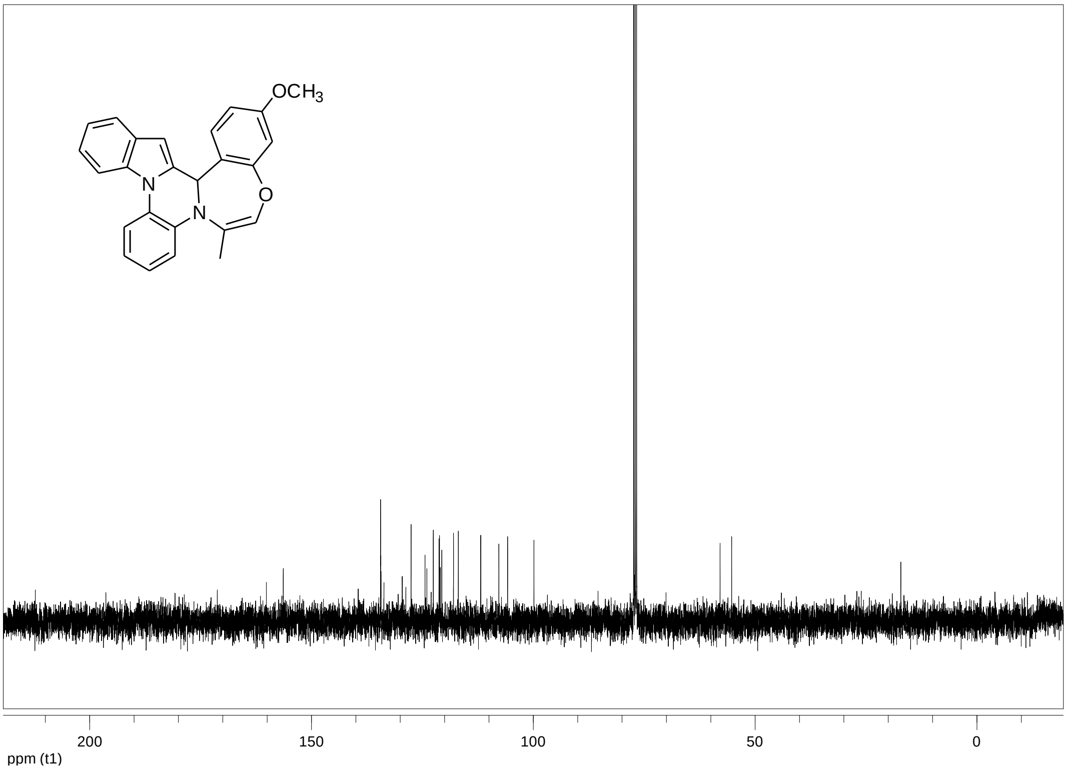

Figure S32. ^{13}C NMR Spectrum of compound **10i**.

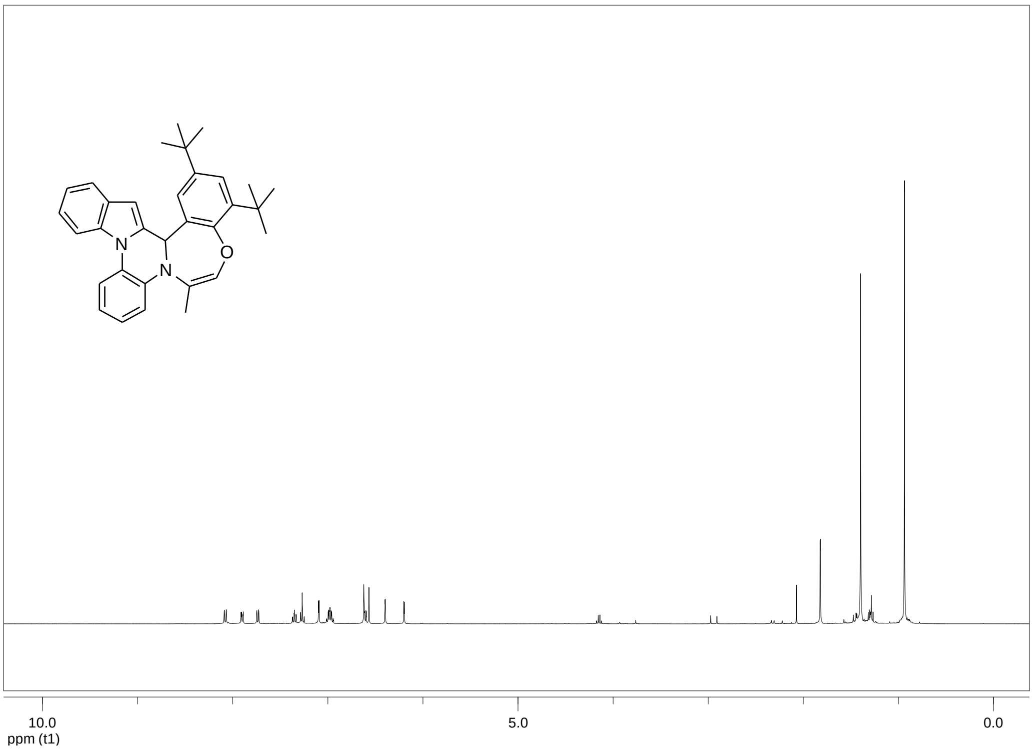

Figure S33. ^{1}H NMR Spectrum of compound **10j**.

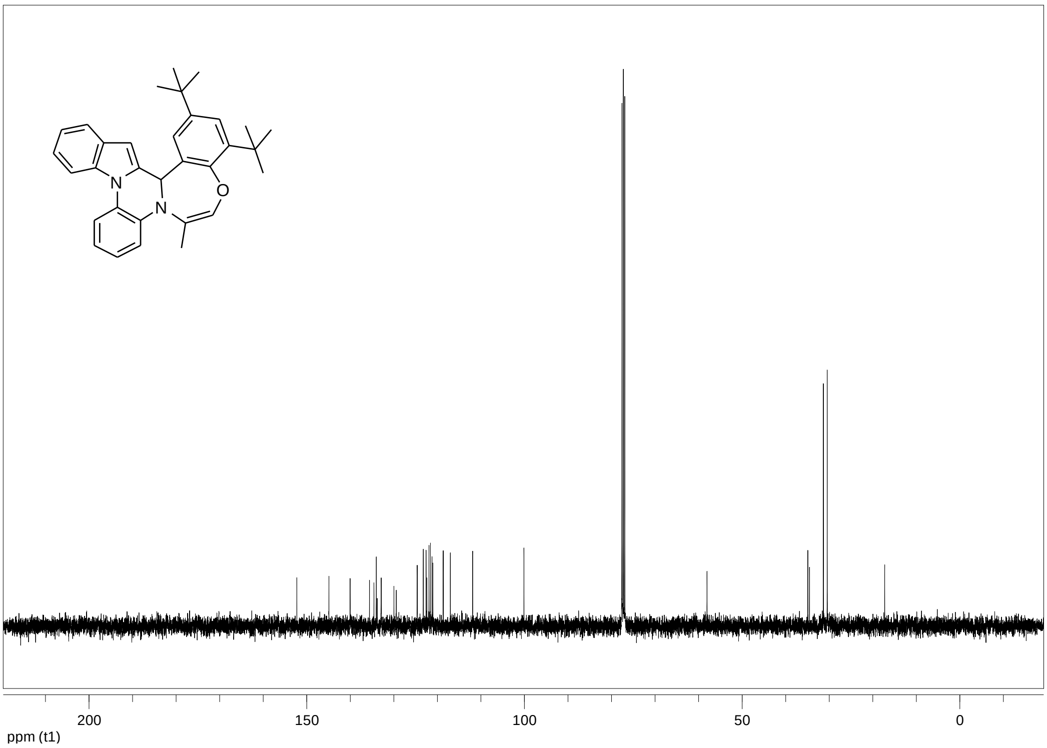

Figure S34. ^{13}C NMR Spectrum of compound **10j**.

References

1. Wanga, Y.; Zhua, Q. Palladium(II)-Catalyzed Cycloamidination via (sp^2)–H Activation and Isocyanide Insertion. *Adv. Synth. Catal.* **2012**, *354*, 1902–1908.

2. An, Z.; Wu, M.; Kang, J.; Ni, J.; Qi, Z.; Yuan, B.; Yan, R. Synthesis of Fused B-Containing Heterocyclic Compounds and Their Relevant Optical Properties. *Eur. J. Org. Chem.* **2018**, 4812–4817.

3. Antilla, J. C.; Klapars, A.; Buchwald, S. L. The Copper-Catalyzed *N*-Arylation of Indoles. *J. Am. Chem. Soc.* **2002**, *124,* 11684–11688.

4. Chandrasekhar, S.; Sultana, S. S.; Yaragorla, S. R.; Reddy, N. R. Copper-Catalyzed N-Arylation of Amines/Amides in Poly(ethylene glycol) as Recyclable Solvent Medium. *Synthesis* **2006**, 839–842.

5. Hans, R. H.; Guantai, E. M.; Lategan, C.; Smith, P. J.; Wanc, B.; Franzblau, S. G.; Gut, J.; Rosenthal, P. J.; Chibale, K. Synthesis, antimalarial and antitubercular activity of acetylenic chalcones. *Bioorg. Med. Chem. Lett.* **2010**, *20,* 942–944.

6. S. A. I. Sharif, E. D. D. Calder, A. H. Harkiss, M. Maduro, A. Sutherland, Synthesis of Allylic Amide Functionalized 2*H*–Chromenes and Coumarins Using a One-Pot Overman Rearrangement and Gold(I)-Catalyzed Hydroarylation. *J. Org. Chem.* **2016**, *81*, 9810−9819.

7. Biju, A. T.; Wurz, N. E.; Glorius, F.; N-Heterocyclic Carbene-Catalyzed Cascade Reaction Involving the Hydroacylation of Unactivated Alkynes. *J. Am. Chem. Soc.* **2010**, *132*, 5970–5971.

8. Khoshkholgh, M. J.; Balalaie, S.; Bijanzadeh, H. R.; Gross, J. H. Intramolecular domino-Knoevenagel-hetero-Diels-Alder reaction with terminal acetylenes. *ARKIVOC* **2009**, *ix*, 114–121.

9. Keskin, S.; Balci; M. Intramolecular Heterocyclization of *O*–Propargylated Aromatic Hydroxyaldehydes as an Expedient Route to Substituted Chromenopyridines under Metal-Free Conditions. *Org. Lett.* **2015,** *17*, 964−967.

10. Vedachalam, S.; Wong, Q. L.; Maji, B.; Zeng, J.; Ma, J.; Liu, X. W. N-Heterocyclic Carbene Catalyzed Intramolecular Hydroacylation of Activated Alkynes: Synthesis of Chromones. *Adv. Synth. Catal.* **2011**, *353*, 219–225.

11. Padmanaban, M.; Biju, A. T.; Glorius, F. Efficient Synthesis of Benzofuranones: N-Heterocyclic Carbene (NHC)/Base-Catalyzed Hydroacylation–Stetter–Rearrangement Cascade. *Org. Lett*. **2011**, *13*, 5624–5627.

12. Muthusamy, S.; Gangadurai, C. ‘‘On water” cascade synthesis of benzopyranopyrazoles and their macrocycles. *Tetrahedron Lett*. **2018**, *59*, 1501–1505.

13. Phan, J.; Ruser, S. M.; Zeitler, K.; Rehbein, J. NHC-Stabilized Radicals in the Formal Hydroacylation Reaction of Alkynes. *Eur. J. Org. Chem*. **2019**, 557–561.
